# Long small RNA76113 targets CYCLIC NUCLEOTIDE-GATED ION CHANNEL 5 to repress disease resistance in rice

**DOI:** 10.1093/plphys/kiad599

**Published:** 2023-11-09

**Authors:** Liyu Zheng, Yiyang Yu, Ying Zheng, Yaxin Wang, Na Wu, Chunhao Jiang, Hongwei Zhao, Dongdong Niu

**Affiliations:** Department of Plant Pathology, College of Plant Protection, Nanjing Agricultural University, and Key Laboratory of Integrated Management of Crop Diseases and Pests, Ministry of Education, Nanjing 210095, China; State Key Laboratory of Biological Interactions and Crop Health, Nanjing Agricultural University, Nanjing 210095, China; Department of Plant Pathology, College of Plant Protection, Nanjing Agricultural University, and Key Laboratory of Integrated Management of Crop Diseases and Pests, Ministry of Education, Nanjing 210095, China; State Key Laboratory of Biological Interactions and Crop Health, Nanjing Agricultural University, Nanjing 210095, China; Department of Plant Pathology, College of Plant Protection, Nanjing Agricultural University, and Key Laboratory of Integrated Management of Crop Diseases and Pests, Ministry of Education, Nanjing 210095, China; State Key Laboratory of Biological Interactions and Crop Health, Nanjing Agricultural University, Nanjing 210095, China; Department of Plant Pathology, College of Plant Protection, Nanjing Agricultural University, and Key Laboratory of Integrated Management of Crop Diseases and Pests, Ministry of Education, Nanjing 210095, China; State Key Laboratory of Biological Interactions and Crop Health, Nanjing Agricultural University, Nanjing 210095, China; Department of Plant Pathology, College of Plant Protection, Nanjing Agricultural University, and Key Laboratory of Integrated Management of Crop Diseases and Pests, Ministry of Education, Nanjing 210095, China; State Key Laboratory of Biological Interactions and Crop Health, Nanjing Agricultural University, Nanjing 210095, China; Department of Plant Pathology, College of Plant Protection, Nanjing Agricultural University, and Key Laboratory of Integrated Management of Crop Diseases and Pests, Ministry of Education, Nanjing 210095, China; State Key Laboratory of Biological Interactions and Crop Health, Nanjing Agricultural University, Nanjing 210095, China; Department of Plant Pathology, College of Plant Protection, Nanjing Agricultural University, and Key Laboratory of Integrated Management of Crop Diseases and Pests, Ministry of Education, Nanjing 210095, China; State Key Laboratory of Biological Interactions and Crop Health, Nanjing Agricultural University, Nanjing 210095, China; Department of Plant Pathology, College of Plant Protection, Nanjing Agricultural University, and Key Laboratory of Integrated Management of Crop Diseases and Pests, Ministry of Education, Nanjing 210095, China; State Key Laboratory of Biological Interactions and Crop Health, Nanjing Agricultural University, Nanjing 210095, China

## Abstract

Small RNAs are widely involved in plant immune responses. However, the role of long small RNAs (25 to 40 nt) in monocot plant disease resistance is largely unknown. Here, we identified a long small RNA (lsiR76113) from rice (*Oryza sativa*) that is downregulated by *Magnaporthe oryzae* infection and targets a gene encoding CYCLIC NUCLEOTIDE-GATED CHANNEL 5 (CNGC5). The *cngc5* mutant lines were more susceptible to *M. oryzae* than the wild type, while knocking down lsiR76113 in transgenic rice plants promoted pathogen resistance. A protoplast transient expression assay showed that OsCNGC5 promotes Ca^2+^ influx. These results demonstrate that OsCNGC5 enhances rice resistance to rice blast by increasing the cytosolic Ca^2+^ concentration. Importantly, exogenous Ca^2+^ application enhanced rice *M*. *oryzae* resistance by affecting reactive oxygen species (ROS) production. Moreover, *cngc5* mutants attenuated the PAMP-triggered immunity response, including chitin-induced and flg22-induced ROS bursts and protein phosphorylation in the mitogen-activated protein kinase cascade, indicating that OsCNGC5 is essential for PAMP-induced calcium signaling in rice. Taken together, these results suggest that lsiR76113-mediated regulation of Ca^2+^ influx is important for PTI responses and disease resistance in rice.

## Teaser

Long small RNA76113 inhibits immune responses in rice (*Oryza sativa*) by suppressing the calcium signaling regulator CYCLIC NUCLEOTIDE-GATED ION CHANNEL 5.

## Introduction

The plant immune system has a dual recognition response mechanism that includes pathogen-associated molecular pattern (PAMP)-triggered immunity (PTI) and effector-triggered immunity (ETI) (Liu et al. 2013). As the first layer of defense for plants to resist the invasion of pathogens, plants recognize conserved PAMPs through pattern recognition receptors (PRRs) to trigger immune responses and limit pathogen invasion. PTI responses include increased intracellular calcium content, reactive oxygen species (ROS) burst, MAPK cascade activation, and PTI-related defense gene expression (Boller and Felix 2009). ETI is based on an immune receptor encoded by the resistance (*R*) genes in plants, and it initiates an immune response by directly or indirectly identifying the toxic effectors secreted by pathogens into the plant cells. As the second layer of defense, ETI is usually concomitant with a hypersensitive response (HR) (Jones and Dangl 2006). Evidence has shown that cyclic nucleotide-gated channels (CNGCs) are implicated in plant PTI/ETI by affecting Ca^2+^ signaling (Tian et al. 2020).

Calcium (Ca^2+^) is a ubiquitous signal in eukaryotes. In plants, Ca^2+^ signaling is involved in diverse processes, including developmental processes; responses to abiotic stresses, such as drought, heat, and wounding; and biotic stimuli, such as immune elicitors (Kadota et al. 2004; Yuan et al. 2017; Moeder et al. 2019). CNGCs are nonselective cation channels that are activated by cyclic nucleotides (cAMP and cGMP). The earlier identified plant CNGCs in barley (*Hordeum vulgare*) were calmodulin (CaM)-binding proteins (Schuurink et al. 1998). Plant CNGCs are part of the signal transduction cascade, which can participate in Ca^2+^ influx to initiate an early defense response to pathogen invasion (Dangl et al. 1996; Hetherington and Brownlee 2004). The Arabidopsis (*Arabidopsis thaliana*) genome encodes 20 CNGC members that are associated with the regulation of development, biotic/abiotic stress responses, and ion homeostasis (DeFalco et al. 2016b). In the *dnd1* or *dnd2* (defense, no death) mutant, Arabidopsis lacking a functional AtCNGC2 or AtCNGC4 results in an impaired hypersensitive response to avirulent pathogens and inhibits the CNGC2-dependent pathogen response signaling cascade through a lack of cAMP-activated inward plasma membrane Ca^2+^ current (Clough et al. 2000; Jurkowski et al. 2004; Ali et al. 2007). CNGC2 and CNGC4 assemble into a functional calcium channel, which is phosphorylated and activated by BOTRYTIS-INDUCED KINASE1 (BIK1) to trigger an increase in cytosolic calcium and calcium-dependent immune responses upon pathogen invasion (Tian et al. 2019). *AtCNGC11* and *AtCNGC12* insertion mutant plants exhibit decreased resistance to infection by the avirulent (but not by the virulent) oomycete pathogen *Hyaloperonospora arabidopsidis* and bacterial pathogen *Pseudomonas syringae*, indicating the positive regulatory role of both *AtCNGC11* and *AtCNGC12* in *R* gene-mediated resistance responses against different pathogens (Moeder et al. 2011). Arabidopsis CNGC19 was also found to be critical for innate immunity against herbivory by activating herbivory-induced Ca^2+^ dense signaling (Meena et al. 2019). In rice (*Oryza sativa*), 16 full-length *CNGC* genes have been identified, and their expression is highly responsive to multiple stimuli, including hormonal, biotic, and abiotic stress (Nawaz et al. 2014). OsCNGC9 is required for disease resistance against rice blast because it is critical in PAMP-triggered defense responses, such as Ca^2+^ influx, ROS burst, and induction of defense-related genes (Wang et al. 2019). Loss of either *OsCNGC14* or *OsCNGC16* reduced cytosolic Ca^2+^ signals induced by temperature stress and reduced survival rates under both heat and chilling conditions (Cui et al. 2020).

Calcium signaling in plants plays a key role in regulating a variety of biological processes. Calcium ion (Ca^2+^) is an important cell signaling molecule that can regulate many physiological and developmental processes through concentration changes. Calcium signaling is involved in the regulation of flowering in plants, affecting the differentiation and opening of flower buds (Furuyama and Dzelzkalns 1999). Plants regulate root hair development through calcium signaling to increase root water and nutrient uptake (Tan et al. 2020). When plants are subjected to adversity (such as salt stress, drought, pathogen attack, etc.), they will produce Ca^2+^ responses to trigger corresponding adaptive responses (Zheng et al. 2013). In the immune response of plants, Ca^2+^ signal plays a key role in signaling after sensing pathogen invasion, triggering a series of defense responses (Jiang and Ding 2023). Ca^2+^ is also involved in plant–microbe symbiosis (Wang et al. 2022). Take plant-microbe symbiosis as an example, Ca^2+^ plays a key role in the symbiotic relationship between plants and beneficial microorganisms such as rhizobia bacteria. In *Medicago-*rhizobial symbiotic relationship, the symbiotic signaling occurs when the plant receptor complex LysM receptor kinase 3 (LYK3)-Nod factor perception (NFP) recognizes lipo-chitooligosaccharide signals from rhizobia bacteria (i.e. Nod factors) (Haney et al. 2011; Kang et al. 2011). Recognition of Nod factors (NF) by plant cells activates Ca^2+^ channels such as CYCLIC NUCLEOTIDE-GATED CHANNEL 15 (CNGC15a, b, c) and DMI1 (DOES NOT MAKE INFECTIONS, originally reported as potassium channels) (Ane et al. 2004; Peiter et al. 2007; Charpentier et al. 2016). Nod factor recognition also activates the Ca^2+^ pump, a membrane Ca^2+^-ATPase 8 (MCA8). As a result, sharp oscillations of cytoplasmic and perinuclear Ca^2+^ occurs, a phenomenon known as Ca^2+^ spikes (Ehrhardt et al. 1996; Wang et al. 2022). Following Nod factors induced Ca^2+^ influx, some Ca^2+^ binding proteins [such as Calcium/Calmodulin-Dependent Protein Kinase (CCaMK)] decode symbiotic Ca^2+^ into downstream phosphorylation events (Gleason et al. 2006). Ca^2+^ and CaM-bound CCaMK phosphorylates transcription factors and induces symbiosis-associated gene expression to initiate nodulation.

Small RNAs are noncoding RNA with a length of 21 to 24 nucleotides (nt). The combination of small RNA and Argonaute (AGO) protein constitutes the RNA-induced silencing complex (RISC), which combines complementary bases with intracellular mRNA to directly cleave or inhibit translation in post-transcriptional gene regulation [post-transcriptional gene silencing (PTGS)] or transcriptional gene silencing (TGS) (Zeng et al. 2003; Bartel 2004; Guo et al. 2010). The earlier found siRNA, nat-siRNAATGB2, reported to regulate plant immunity was derived from the overlapping region of an antisense transcript pair and highly specifically induced by the bacterial pathogen *Pseudomonas syringae* pv. *tomato* (*Pst*) carrying the effector avrRpt2 (Katiyar-Agarwal et al. 2006). It inhibits a negative regulator of plant defense, a pentapeptide-like protein, and promotes ETI only when infected.

Many small RNAs longer than 24 nt are also found in animals and plants. scnRNAs are found in *Tetrahymena* and are approximately 27 to 30 nt in length. They cooperate with the AGO protein to participate in DNA clearance (Hock and Meister 2008). piRNAs, with a length of approximately 26 to 31 nt, are the largest type of small RNAs expressed in animal cells. piRNAs can specifically bind to PIWI subfamily proteins in the AGO protein family and are required for spermatogenesis in humans and nematodes (Wang and Reinke 2008). Plant sRNAs can be divided into two groups, microRNAs (miRNAs) and small interfering RNAs (siRNAs), based on their synthetic processes and precursor structures (Chen 2009). Plant siRNAs are further classified into trans-acting siRNAs (ta-siRNAs), natural antisense transcript-derived siRNAs (nat-siRNAs), heterochromatic siRNAs (hc-siRNAs), and long siRNAs (lsiRNAs) (Huang et al. 2019b). Plant endogenous lsiRNAs with lengths of approximately 30 to 40 nt were identified in Arabidopsis under the induction of bacterial pathogens or specific growth conditions (Katiyar-Agarwal et al. 2007). AtlsiRNA1, which is specifically induced by *Pst* (*avrRpt2*), contributes to ETI by silencing *RNA-binding domain abundant in Apicomplexans* (*AtRAP*), a negative regulator of plant basal defense responses against *Pst* (*avrRpt2*) infection (Katiyar-Agarwal et al. 2007). In a previous study, we showed that *Rhizoctonia solani* infection alters the expression of lsiRNAs in rice, thus demonstrating that fungal pathogens can also induce plant lsiRNA (Niu et al. 2016). However, the exact immune function of lsiRNAs in monocot plants remains largely unknown.

Rice blast is one of the most serious diseases of rice, and it is caused by *Magnaporthe oryzae*. The mining and exploitation of rice resistance genes are extremely important for the control of rice blast fungi. However, resistance genes are easily lost under selective pressure, so further research on the rice blast resistance mechanisms is particularly important for the development of reliable disease control methods. In this study, long small RNAs that response to *M. oryzae* treatment were obtained from rice. In particular, we found that lsiR76113 strongly suppressed and negatively regulated the cyclic nucleotide-gated ion channel (*OsCNGC5*). The lsiR76113 silent mutant enhances disease resistance, whereas the *oscngc5* mutant shows higher susceptibility to *M. oryzae* infection. In addition, OsCNGC5-mediated Ca^2+^ influx is required for immune responses, such as ROS burst. Moreover, exogenous Ca^2+^ treatment impaired ROS burst and blast resistance in the *cngc5* mutant. Further analysis indicated that OsCNGC5 activates the PTI response in rice.

## Results

### Characterization of lsiRNAs in response to *M. oryzae* infection

To study the role of lsiRNA in the process of rice defense against *M. oryzae*, we compared and analyzed the difference in osa-lsiRNA expression levels between a *M. oryzae* spraying treatment (1 × 10^5^ spores·mL^−1^) and the control treatment (water) at 24 h post inoculation. A total of 40,986,239 and 60,880,620 reads were obtained from the control and pathogen-infected groups, respectively (Table 1). Among these, 34,233,972 and 44,408,614 reads, which accounted for 83.53% and 72.42% of the total reads, respectively, were successfully mapped into the rice genome (Table 1). The mapped reads were further screened using the Rfam database (http://xfam.org/) to filter out ribosomal RNA (rRNA), transfer RNA (tRNA), and small nucleolar RNAs (snoRNAs) that interfered with our analysis, resulting in 14,710,149 and 31,847,614 reads (Table 1). These lsiRNAs were used for further classification based on their origin in the rice genome. Of the lsiRNAs, 86.8% were derived from transposable elements (TEs) and repeat regions, 4.26% were derived from intergenic regions, and 3.45% were derived from promoter regions. Less than 1% (0.12%) originated from the miRNA-coding region (Supplemental Fig. S1, A and B). The identified lsiRNA differentially expressed in *M*. *oryzae* infected rice were listed in Supplemental Table S1.

**Table 1.** Summary of osa-lsiRNA in *Magnaporthe oryzae-*infected samples

Read categories	Mock	(%)	Infected	(%)
Total reads	40,986,239	100	60,880,620	100
Rice reads (mapped to rice genome)	34,233,972	83.53	44,088,614	72.42
Aligned to Rfam (rRNA, tRNA, snRNA, and snoRNA)	19,523,823	47.64	12,241,000	20.11
Rice sRNA (total reads)	14,710,149	35.89	31,847,614	52.31

**Figure 1.**
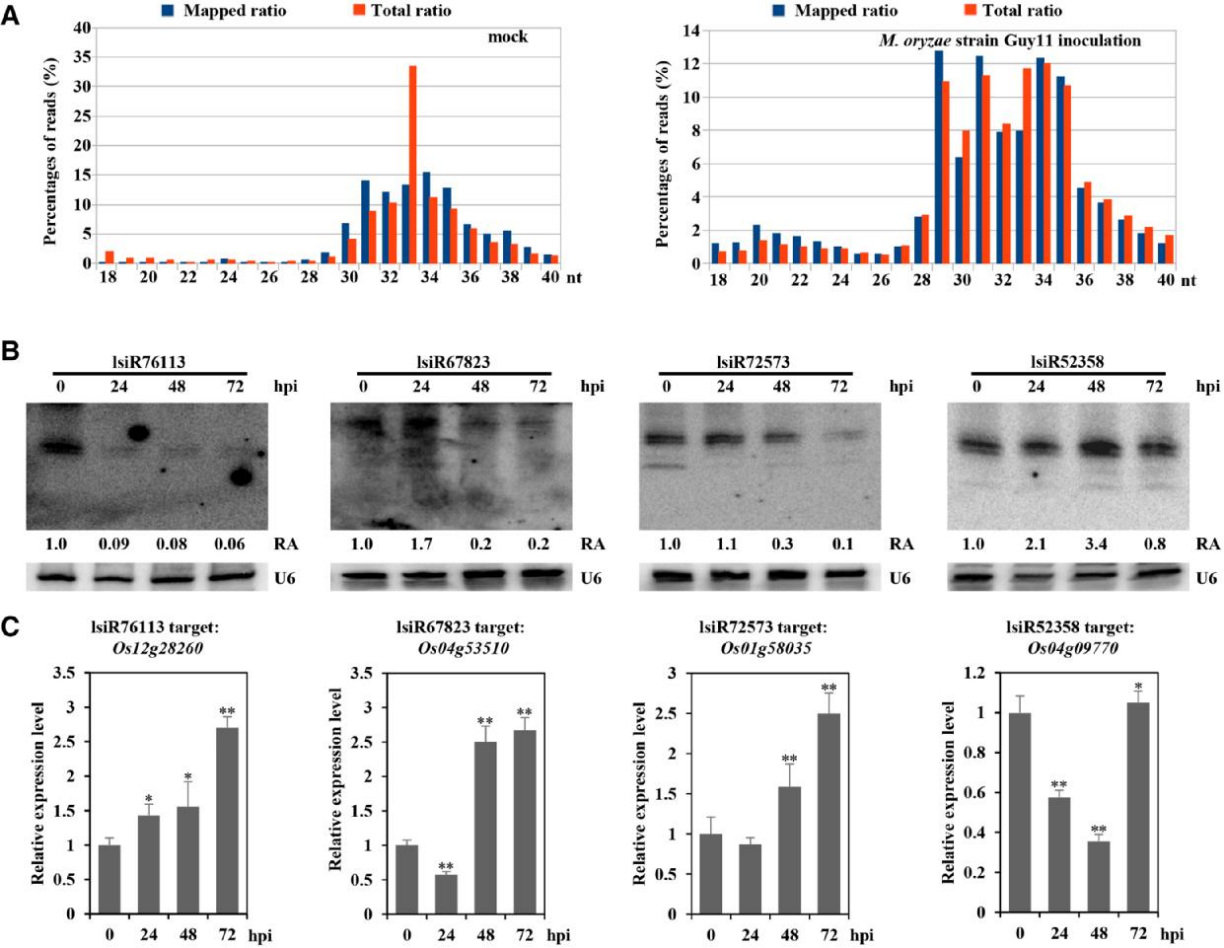
Expression of lsiRNAs is altered upon *M*. *oryzae* treatment. **A)** Length distributions of differentially expressed long small RNAs with (left panel) or without (right panel) *M. oryzae* strain Guy11 inoculation. **B)** Two-week-old rice seedlings were inoculated with Guy11 spores (1 × 10^5^ spores mL^−1^), and total RNA was extracted at the indicated time points. Northern blotting was performed to verify the expression level of the lsiRNAs (lsiR76113, lsiR67823, lsiR72573, and lsiR52358) identified as differentially expressed in the transcriptional sequencing. U6 was used as a loading control. LsiRNA, long siRNA; U6, U6 small nuclear RNA (snRNA). RA, relative abundance values represent the corresponding RNA normalized to U6. hpi, hours post-infection. **C)** Comparative quantitative real-time RT-qPCR verification of predicted targets of the differentially expressed lsiRNAs. The values are presented as ± SD (*n* = 3 replicates). The Student's t-test analysis indicates a significant difference (*P < 0.05, **P < 0.01). Three independent biological experiments were carried out, and all three repetitions showed similar results.

Subsequently, we analyzed the length distribution of the filtered sRNAs. Among them, lsiRNAs with a length ranging from to 28 to 40 nt accounted for more than 85% of the total while sRNAs with no more than 27 nt accounted for less than 5%. sRNA with a length of 31 to 35 nt accounted for more than half of the total reads of the long-stranded sRNAs (Fig. 1A).

### *Magnaporthe oryzae* infection substantially suppressed lsiR76113 expression

The targets of lsiRNAs with substantially altered expression levels were predicted (http://www.zhaolab.org/psRNATarget/) (Table 2). We performed northern blotting to verify the expression levels of four sRNAs (lsiR76113, lsiR67823, lsiR72573, and lsiR52358), and the most substantial differences in expression were based on the sequencing data. The results showed that the changes in sRNAs in rice after the pathogen treatment were consistent with the sequencing results (Fig. 1B), and the downward trend of lsiR76113 was particularly remarkable. The expression levels of the predicted target genes were determined using RT-qPCR (Fig. 1C). Consistent with the downward trend of lsiR76113, the expression level of its target gene *Os12g28260* increased significantly under *M. oryzae* infection (Fig. 1C). Likewise, the expression profiles of *Os04g53510*, *Os01g58035*, and *Os04g09770* exhibited a reverse trend to those of their corresponding regulatory sRNAs (Fig. 1C).

**Table 2.** Differentially expressed rice lsiRNAs and their potential target genes

ID	Length (nt)	Target gene	Annotation	Score	Alignment
lsiR76113	29	Os12g28260	Cyclic nucleotide-gated ion channel	0	:::::::::::::::::::::::::::::
		Os06g29350	Myosin	3	:: : :::::::::: :.::::::…
lsiR72573	29	Os01g58035	Expressed protein	0	:::::::::::::::::::::::::::::
lsiR67823	28	Os04g53510	OsFBL20—F-box domain and LRR containing protein	3	:::::::::::::: :.: :::
lsiR52358	30	Os04g09770	Ser/Thr protein kinase	2	:..::::::::: ::::::
lsiR69852	30	Os04g16775	Conserved hypothetical protein	0	:::::::::::::::::::::::::::
		Os07g25022	Conserved hypothetical protein	0	::::::::::::::::::::::::::::
		Os08g15264	Conserved hypothetical protein	0	::::::::::::::::::::::::::::
		Os09g24408	Conserved hypothetical protein	0	::::::::::::::::::::::::::::
lsiR44650	29	Os02g30150	Disease resistance protein	3	.: :.:.::::::::.::: :.::::
lsiR72009	28	Os01g27150	Cullin	3	: :. .:.:.:::::::.:.:::.: ::
		Os08g01810	Matrix attachment region binding protein	3	:.::::::.. ::::::::.
lsiR3203	29	Os08g12750	Serine/threonine-protein kinase HT1	3	: .::::::::::.:::::
lsiR73261	28	Os05g06270	Zinc finger, C3HC4 type domain containing protein	2	.: :::.:::::::::.:::
lsiR97570	29	Os11g18670	Ubiquitin family protein	3	::::: ::: ::: :::::::
lsiR90051	29	Os01g04490	Ser/Thr protein kinase	2.5	:::.:::::::::::::: :
lsiR52358	30	Os04g09770	Ser/Thr protein kinase	2	:..::::::::: ::::::
lsiR84848	30	Os04g54002	Serine/threonine-protein kinase receptor precursor	3	::: :::::.:: :: :.::::::
lsiR95113	30	Os01g22990	Acidic leucine-rich nuclear phosphoprotein 32-related protein 1	2.5	: :. :: ::::::::: ::::.:
lsiR48059	29	Os02g54020	DEAD-box ATP-Dependent RNA helicase	2	:: ::::::::::::::::: ::::

### Expression level of *OsCNGC5* is inhibited by lsiR76113

**Figure 2.**
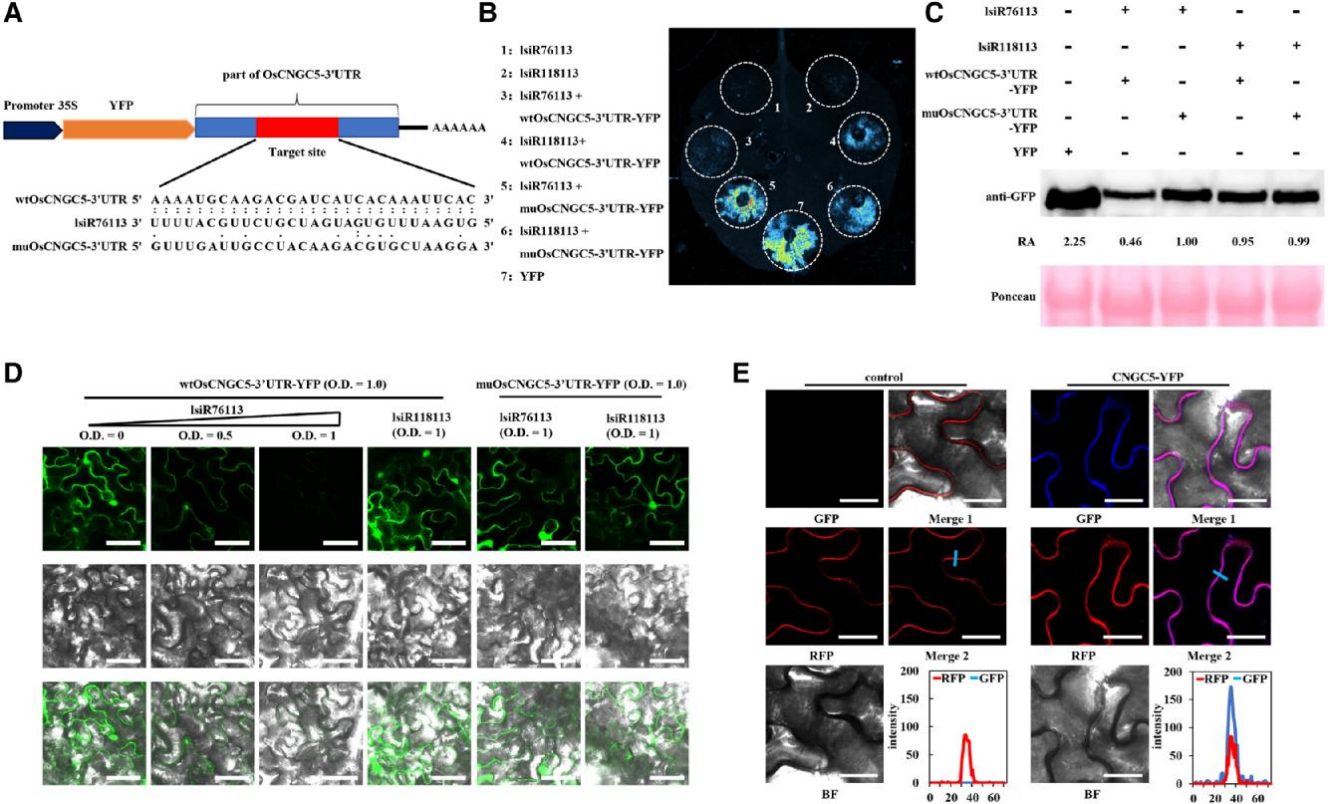
Validation of the target of lsiR76113 and sublocation of protein OsCNGC5. **A)** The target sequence was cloned into pBIN-3HA vector with YFP protein (pBIN-3HA-YFP-CNGC5-3′UTR-wt), and the mutated target sequence (pBIN-3HA-YFP-CNGC5-3′UTR-mu) was used as a negative control. lsiR76113 was expressed in pBIN-3flag vector (pBIN-3FLAG-lsiR76113). YFP, yellow fluorescent protein; 3′UTR, 3′ untranslated region. “:”, standard Watson–Crick conformations. “.”, wobble U-G conformations. wt, wild type; mu, mutant. **B)** Transient co-expression assays were performed by infiltrating 3-week-old *N. benthamiana* plants with *Agrobacterium tumefaciens* GV3101 (OD600 = 1) as described. The fluorescence image was captured with Fusion-FX7 Spectra. **C)** Leaf tissue was collected 48 hpi, and protein expression was detected by immunoblot analysis. “±”, agrobacterium infiltration with or without this fragment. RA, relative abundance. **D)** Transient co-expression assay was performed by infiltrating *N. benthamiana* with *Agrobacterium tumefaciens* GV3101 at the indicated concentration. The intensity of fluorescence was examined with Olympus BX43. pBIN-3HA-YFP-wt/muOsCNGC5-3′UTR and pBIN-3FLAG-lsiR76113 were used in **(B–D)**. Scale bars, 50 *μ*m. O.D., optical density. **E)** Confocal microscope showing the sub-localization results of OsCNGC5-YFP protein and FM4-64 (plasma membrane) staining. Use ImageJ software to calculate the fluorescence intensity values of GFP and RFP on the path of the blue line in merge 2, and compile it into a line chart based on the data. RFP, red fluorescent protein; GFP, green fluorescent protein. Scale bars, 25 *μ*m.

To further verify whether lsiR76113 inhibits the expression of *Os12g28260* (*OsCNGC5*), a transient expression analysis in *Nicotiana benthamiana* was conducted. The target site and its upstream and downstream sequences (part of *OsCNGC5* 3′ untranslated region) were cloned to construct a YFP fusion protein under the control of the 35S promoter (Fig. 2A), and the random mutated target sequence was used as a negative control. lsiR76113 and lsiR118113 (a negative control) were expressed using the pBIN-3flag vector. A substantial decline of YFP fluorescence signal was detected during the transient co-expression of lsiR76113 and YFP-wtOsCNGC5-3′UTR, while a strong YFP signal was observed during the coexpression of lsiR76113/YFP-muOsCNGC5-3′UTR group, lsiR118113/YFP-wtOsCNGC5-3′UTR group, and lsiR118113/YFP-muOsCNGC5-3′UTR group, suggesting that lsiR76113 can inhibit the expression of *OsCNGC5* by specifically recognizing the target sequence (Fig. 2B). Western blotting results showed that the expression of lsiR76113 substantially reduced the protein content of wild-type CNGC5 in leaves but had no substantial effect on other groups, which further verified the targeted inhibition of lsiR76113 at the protein level (Fig. 2C). The intensity of YFP in *N*. *benthamiana* expressing YFP-wtOsCNGC5-3′UTR and YFP-muOsCNGC5-3′UTR was also observed with confocal microscope. Wild-type OsCNGC5 and mutated OsCNGC5 presented high accumulation of YFP fluorescence (Fig. 2D). However, YFP fluorescence was reduced when lsiR76113 was coexpressed with YFP-wtOsCNGC5-3′UTR in an optical density (OD)-dependent manner. In contrast, lsiR76113 co-expression with YFP-muOsCNGC5-3′UTR did not affect YFP protein accumulation. On the other hand, lsiR118113 did not affect YFP protein accumulation of YFP-wtOsCNGC5-3′UTR or YFP-muOsCNGC5-3′UTR (Fig. 2D). These results indicated that lsiR76113 targeted and inhibited the expression of *OsCNGC5*.

### OsCNGC5 is localized to the plasma membrane

CNGC family proteins are generally considered to be located in the plasma membrane, such as AtCNGC2 (Chin et al. 2013; Chou et al. 2016), AtCNGC12 (DeFalco et al. 2016a), AtCNGC14 (Shih et al. 2015), AtCNGC17 (Ladwig et al. 2015), and AtCNGC18 (Zhou et al. 2014; Gao et al. 2016). However, some members are located in other regions, such as MtCNGC15 located in endoplasmic reticulum (ER) and inner nuclear envelope (INE) (Charpentier et al. 2016), AtCNGC19 located in Vacuole (Yuen and Christopher 2013).

FM4-64 is a kind of lipophilic dye that can fluorescently label plasma membrane. To determine the subcellular localization of OsCNGC5, we referred to previous methods (Tian et al. 2015). Fluorescence signals of CNGC5-YFP protein (GFP) and FM4-64 staining (RFP) were observed by confocal microscopy. The results showed that the OsCNGC5 protein was a protein localized to the cell membrane (Fig. 2E).

### lsiR76113 suppresses the rice immune to plant diseases

To test whether lsiR76113 participates in *M. oryzae-*triggered immune responses by targeting *OsCNGC5*, we used a short tandem target mimic (STTM), a method of inhibiting the expression level of small RNAs (Tang et al. 2012), to construct lsiR76113 knock-down rice lines and the CRISPR method to create transgenic *cngc5* mutant lines (Supplemental Fig. S2). The relative expression of lsiR76113 was significantly reduced in the STTM76113-A and STTM76113-F mutant lines (Supplemental Fig. S2A). Sequences of the target genes in transgenic rice lines were analyzed. Eight nucleotides are missing in *cngc5-f*, and one nucleotide is inserted in *cngc5-1c* (Supplemental Fig. S2B). Both mutations are located in the exon and result in frameshift mutations. These results indicate the successful construction of transgenic rice of lsiR76113 knock-down and *cngc5-1c* mutant. In lsiR76113 knock-down mutant rice, it was found by RT-qPCR that the transcription level of a potential target (*OsMyosin*) of lsiR76113 was not regulated by lsiR76113, while the other one, *OsCNGC5*, was significantly increased (Supplemental Fig. S3). Therefore, we speculated that lsiR76113 may mainly play a role in rice through suppressing *OsCNGC5*.

**Figure 3.**
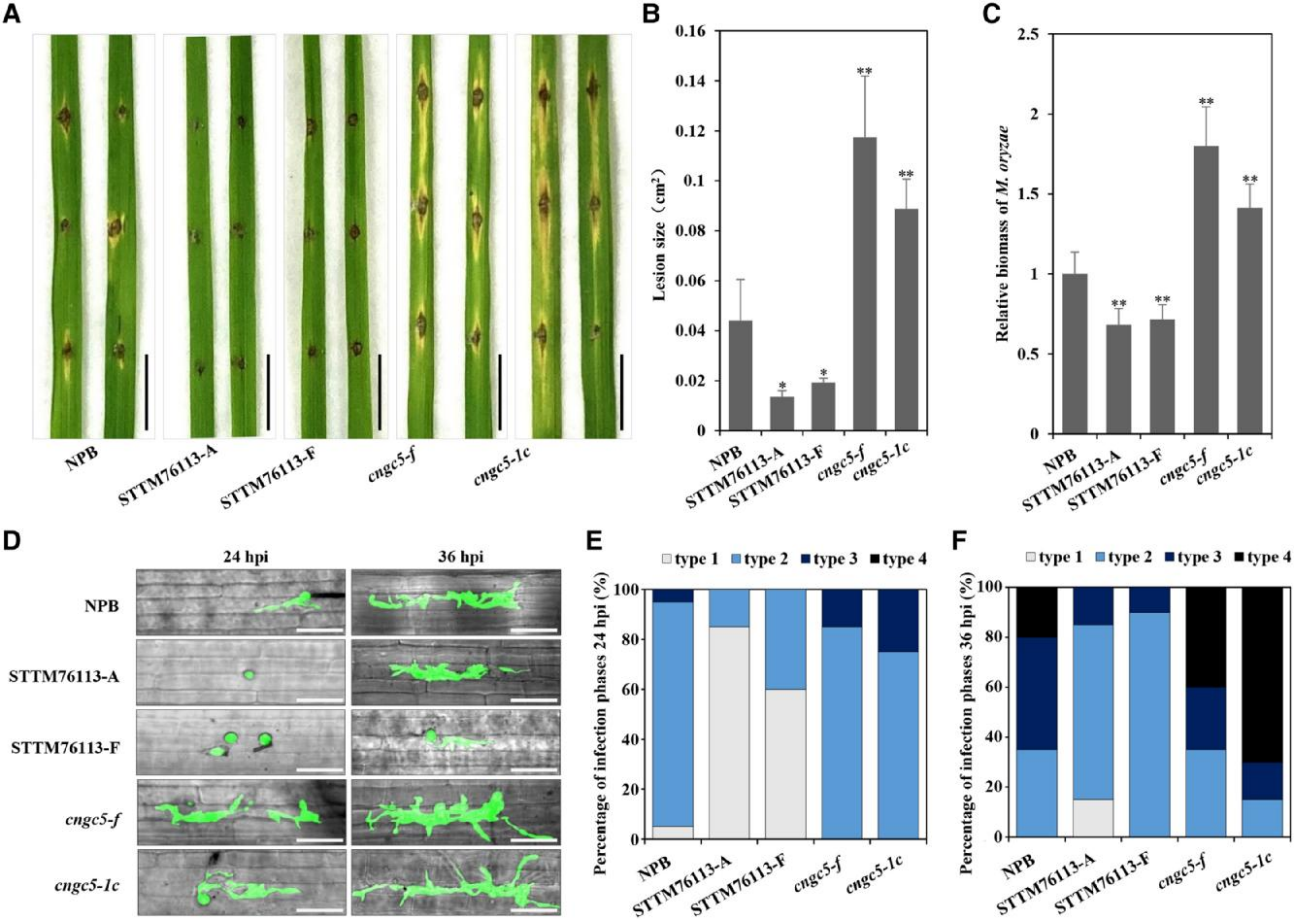
Mutation of *OsCNGC5* leads to enhanced susceptibility to *M. oryzae*. **A)** Disease phenotypes of the leaves of *M. oryzae*-infected NPB (*Oryza sativa* subsp*. japonica*), lsiR76113 knock-down (STTM76113-A/F, short tandem target mimic (STTM) technology to block the functions of lsiR76113), and *cngc5* mutant plants. Leaves were detached, and the *M. oryzae* spore suspension (1 × 10^5^ spores mL^−1^) was drop-inoculated at the wound sites created with a sterile needle. Scale bars, 1 cm. **B)** Lesion size was measured with ImageJ software. **C)** Relative biomass of *M. oryzae* was measured by qPCR. The values are presented as means ± SD (B, *n* = 4 samples; C, *n* = 3 replicates). The Student's t-test analysis indicates a significant difference (*P < 0.05, **P < 0.01). Three independent biological experiments were carried out, and all three repetitions showed similar results. **D)** For spray inoculation, collected spores of the eGFP-labeled *M*. *oryzae* Zhong-1 were inoculated onto the three-leaf-stage seedlings at the concentration of 1 × 10^5^ spores mL^−1^. hpi, hours post-infection. Scale bars, 25 *μ*m. **E, F)** Calculation the number of leaf lesions at **(E)** 24 hpi (*n* = 20 samples) and **(F)** 36 hpi (*n* = 20 samples). Type 1: appressorium formation, Type 2: appressorium infecting a first cell, Type 3: appressorium extending to a second cell, and Type 4: appressorium extending to a third or more cells.

Disease resistance of the transgenic rice lines was tested based on *M. oryzae* punch inoculation. Significantly smaller lesions emerged on the leaves of lsiR76113 knock-down lines compared with those of the *O. sativa* subsp. *japonica* NPB line (Fig. 3, A and B), indicating that the knock-down of lsiR76113 promoted rice immunity to *M. oryzae*. The relative biomass of *M. oryzae* in STTM76113 mutants was also significantly lower than that in NPB (Fig. 3C). In contrast, *cngc5* mutant plants developed significantly larger lesions than NPB and lsiR76113 knock-down lines (Fig. 3, A and B). Similarly, the mutations in *cngc5* resulted in increased relative biomass of *M. oryzae* in rice leaves (Fig. 3C).

We further used an eGFP-labeled *M*. *oryzae* to observe pathogen infection at different time points to explore the details of lsiR76113- and *OsCNGC5*-mediated resistance. Twenty-four hours after pathogen inoculation, aspersorium formation and infection development of the pathogen in lsiR76113 knock-down lines were substantially slower than those in NPB, and the developed *M. oryzae* mycelium in the lsiR76113 knock-down lines was also lower than that in NPB at 36 hpi (Fig. 3D). Quantitative analysis of the pathogen infection also revealed that at 24 hpi, most of the pathogens infecting lsiR76113 knock-down lines were at the onset of appressorium formation while more than 90% of the aspersorium in NPB had infected the first host cell (Fig. 3E). The pathogen infection stage at 36 hpi in the lsiR76113 knock-down lines also lagged substantially behind that of NPB (Fig. 3F). These results suggest that lsiR76113-mediated disease resistance is involved in the early immune response of plants to pathogens. In contrast, at 24 hpi, the appressorium formation and disease progression of *M. oryzae* in *cngc5* mutant plants substantially exceeded those in the NPB wild-type and lsiR76113 knock-down lines (Fig. 3, D and E). Rapid disease progression in the *cngc5* mutant lines was also observed at 36 hpi, indicating that *OsCNGC5* positively regulates plant immunity. Taken together, these results suggest that lsiR76113 negatively regulates the early immune response of plants against *M. oryzae* by inhibiting *OsCNGC5*.

To test the immunity mediated by OsCNGC5 besides rice blast resistance, we tested the resistance of STTM76113-A, NPB and *cngc5*-*1c* to rice sheath blight caused by *R. solani* and bacterial blight caused by *Xanthomonas oryzae* pv. *oryzae* (*Xoo*) (Supplemental Figs. S4 and S5). The results showed that the mutation on *cngc5* reduced the resistance of rice to sheath blight and bacterial blight, while the knock-down mutant of lsiR76113 showed higher resistance to *R. solani* and *Xoo* than NPB. Therefore, our results demonstrated that OsCNGC5 has the potential to confer broad-spectrum resistance against plant diseases. Plant reverses inhibition of OsCNGC5 by reducing the expression of lsiR76113 to enhance the immunity to fungal pathogens *R. solani* and bacterial pathogen *Xoo*.

### lsiR76113 suppresses Ca^2+^ influx and H_2_O_2_ accumulation by inhibiting *OsCNGC5*

**Figure 4.**
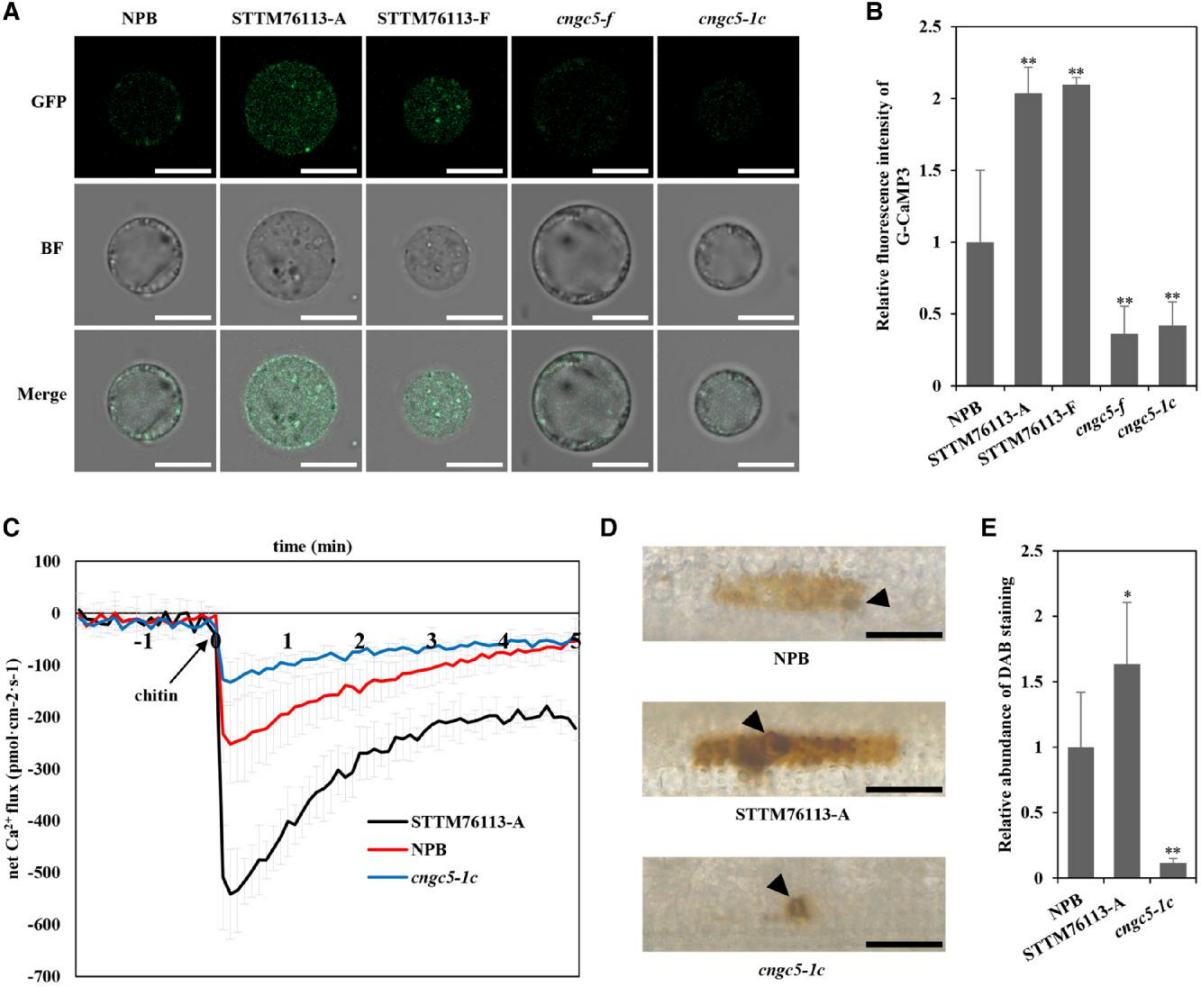
lsiR76113 inhibits Ca^2+^ influx and H_2_O_2_ accumulation by inhibiting the function of OsCNGC5. Microscopy of cytosolic Ca^2+^ in rice protoplast. **A)** Protoplast of indicated lines were transformed with an equal amount of Ca^2+^ reporter G-CaMP3 vector to detect the concentration of cytosolic Ca^2+^. The pictures were taken with Zeiss LSM980 with an Airyscan2 confocal microscope. The figure shows a representation of 6 photos. BF, bright field; GFP, green fluorescent protein. Scale bars, 10 *μ*m. STTM76113-A/F, (lsiR76113 knock-down mutants, short tandem target mimic (STTM) technology to block the functions of lsiR76113). **B)** Intensity of fluorescence was measured with ImageJ software. Asterisks indicate significant differences. **C)** Comparison of Ca^2+^ influx in root tip cells of corresponding lines. The values are presented as means ± SD (*n* = 6 samples). The Student's t-test analysis indicates a significant difference (*P < 0.05, **P < 0.01). Three independent biological experiments were carried out, and all three repetitions showed similar results. **D)** Three-leaf-stage seedlings were spray-inoculated with *M. oryzae* spores at a concentration of 1 × 10^5^ spores mL^−1^. Reactive oxygen species (ROS) burst in rice leaves at 36 hpi was tested by 3,3′-diaminobenzidine (DAB) staining. Scale bars, 50 *μ*m. **E)** Quantification of DAB leaf sheath staining (the gray value on the indicated path of the black arrow) in different strains with ImageJ software in **(D)**. The values are presented as means ± SD (*n* = 5 samples). The Student's t-test analysis indicates a significant difference (*P < 0.05, **P < 0.01). Three independent biological experiments were carried out, and all three repetitions showed similar results.

CNGCs are a family of ion transport channels in plant cell membranes (Jammes et al. 2011). OsCNGCs participate in the influx of Ca^2+^, which is implicated in the early immune response of plants (Hetherington and Brownlee 2004). To determine whether *OsCNGC5* participates in Ca^2+^ influx, the GFP-based fluorescent Ca^2+^ indicator G-CaMP3 was used in a protoplast transient expression assay to detect the concentration of cytosolic Ca^2+^ by confocal microscope (Tian et al. 2009). Compared with the control group, GCaMP3 can indeed make the rice protoplasts emit fluorescent signals, which indicates that this method can be used in cytoplasmic Ca^2+^ measurement (Supplemental Fig. S5). Based on this, we found that a higher GFP fluorescence signal was observed in lsiR76113 knock-down lines than in NPB protoplasts (Fig. 4, A and B), whereas the fluorescence signals in *cngc5-f* and *cngc5-1c* were weaker than those in NPB, STTM76113-A, and STTM76113-F protoplasts (Fig. 4, A and B). These results suggest that lsiR76113 targets *OsCNGC5* and negatively regulates the concentration of cytoplasmic Ca^2+^.

We speculate that the difference in the cytoplasmic Ca^2+^ concentration of rice is caused by the Ca^2+^ influx rate, so we used Non-invasive Micro-test Technology (NMT) to detect the Ca^2+^ uptake rate of different transgenic rice seedling. We found that NPB strain showed stronger Ca^2+^ influx than *cngc5-1c* mutant and weaker than lsiR76113 knock-down lines caused by chitin treatment (Fig. 4C). This indicates that OsCNGC5 can promote Ca^2+^ influx and increase cytoplasmic Ca^2+^ concentration. As we know that the cytoplasmic Ca^2+^ concentration is critical for regulating the production of ROS in the early stages of plant immunity (Wang et al. 2019). So, we performed 3, 3′-diaminobenzidine (DAB) staining to detect the level of ROS production in transgenic and wild-type rice at 36 h post *M. oryzae* inoculation (1 × 10^5^ spores·mL^−1^). Compared with the wild type, the *cngc5-f* and *cngc5-1c* mutants produced less ROS while the STTM76113-A and STTM76113-F mutants produced more ROS (Fig. 4, D and E). These results indicated that *OsCNGC5* enhances ROS accumulation by promoting Ca^2+^ influx and increasing the concentration of cytoplasmic Ca^2+^.

### Exogenous Ca^2+^ application increase disease resistance in rice

To further verify the role of the cytosolic Ca^2+^ concentration in *OsCNGC5*-mediated plant immunity, we supplied rice leaves with a gradient concentration of external Ca^2+^ and observed the resistance of the plants to rice blast. With an external Ca^2+^ application of 0 to 1.5 mm, the resistance of NPB to *M*. *oryzae* was positively related to the supplemented Ca^2+^ in a concentration-dependent manner (Supplemental Fig. S6). So 1.5 mm Ca^2+^ was chosen as the experimental concentration.

**Figure 5.**
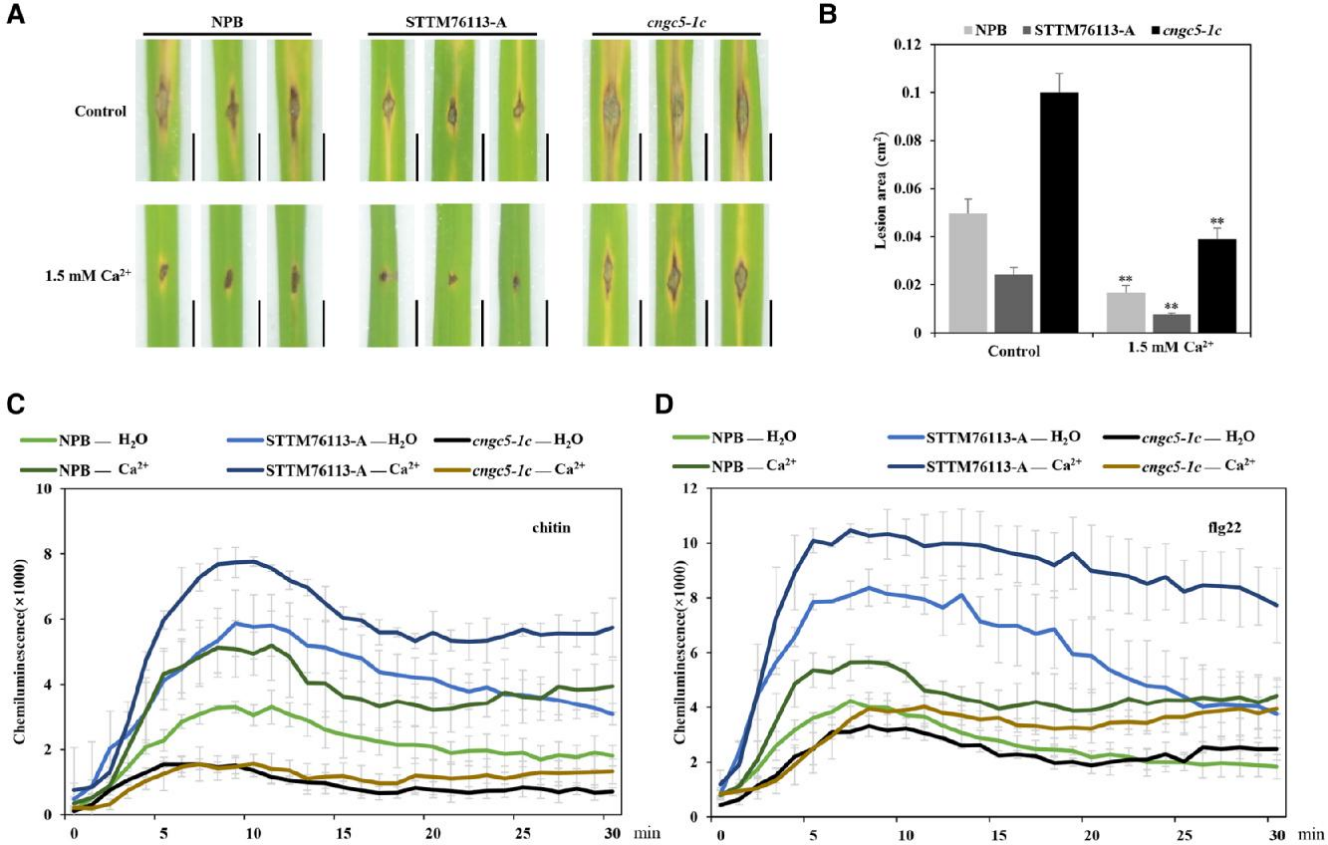
Exogenous Ca^2+^ increase disease resistance in rice. **A)** Disease phenotypes on the leaves of *M. oryzae*-infected NPB (*Oryza sativa* subsp*. japonica*) wild-type, lsiR76113 knock-down (STTM76113-A, short tandem target mimic (STTM) technology to block the functions of lsiR76113), and *cngc5* mutant plants supplemented with 0 or 1.5 mm exogenous Ca^2+^. Scale bars, 0.5 cm. **B)** Lesion size measured with ImageJ software of NPB wild-type, lsiR76113 knock-down and *cngc5-1c* mutant leaves supplemented with 0 or 1.5 mm exogenous Ca^2+^. The values are presented as means ± SD (B, *n* = 3 samples). The Student's t-test analysis indicates a significant difference (*P < 0.05, **P < 0.01). Three independent biological experiments were carried out, and all three repetitions showed similar results. **C, D)** Chitin **(C)** or flg22 **(D)** triggered reactive oxygen species (ROS) burst in the NPB wild-type, lsiR76113 knock-down, and *cngc5-1c* mutant plants with or without 1.5 mm exogenous Ca^2+^ supplementation.

By applying exogenous Ca^2+^, we found that the disease resistance of different strains (including *cngc5-1c* mutant) was improved (Fig. 5, A and B), indicating that OsCNGC5 may merely be one of the regulatory factors of Ca^2+^ influx, and other CNGC families may be involved in Ca^2+^ influx. We further tested the ROS burst of different strains to chitin and flg22 treatment under the condition of exogenous application of 1.5 mm Ca^2+^. We found that 76,113 knock-down strain had the strongest ROS burst, and *cngc5* knock-out strain had the weakest ROS burst (Fig. 5, C and D). These results indicate that the exogenous application of Ca^2+^ enhances the disease resistance response in rice.

### lsiR76113 suppresses rice immunity through PTI pathway

**Figure 6.**
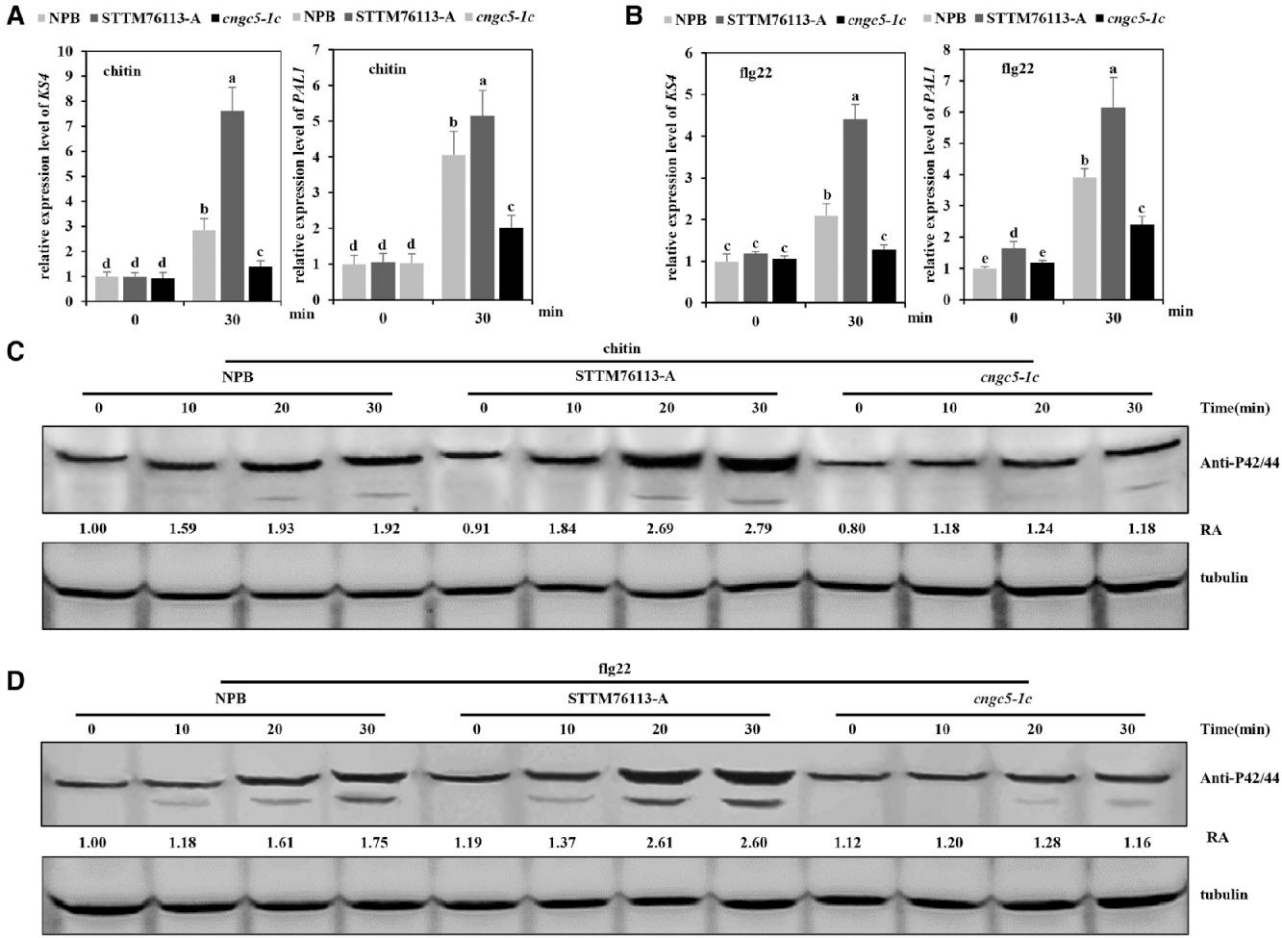
*OsCNGC5* enhances rice PTI response. **A, B)** Relative expression level of *KS4* and *PAL1* in NPB wild-type, lsiR76113 knockdown (STTM76113-A, short tandem target mimic (STTM) technology to block the functions of lsiR76113) and *cngc5* mutant plants after chitin **(A)** or flg22 **(B)** treatment was detected by RT-qPCR. The values are presented as means ± SD (A/B, *n* = 3 replicates). The Student's t-test analysis indicates a significant difference. Lowercase letter (a/b/c/d/e) indicates the significance level α = 0.05. Those with the same letters indicate that the difference is not significant. Three independent biological experiments were carried out, and all three repetitions showed similar results. **C, D)** Protein phosphorylation of the mitogen-activated protein kinase (MAPK) cascade was detected in NPB wild-type, lsiR76113 knockdown and *cngc5* mutant plants following chitin **(C)** or flg22 **(D)** treatment. RA, relative abundance.

To further explore the role of lsiR76113 in plant immunity, we measured the expression level of PTI relative gene and protein phosphorylation in the mitogen-activated protein kinase (MAPK) cascade after chitin and flg22 treatment in the NPB, STTM76113-A mutant and *cngc5-1c* mutant plants. The expression of PTI-related genes (*OsKS4* and *OsPAL1*) was significantly higher in the STTM76113-A mutant than in the wild-type upon chitin and flg22 treatment, whereas the *cngc5* mutant presented repressed PAMP-triggered defense-related gene expression compare with wild-type (Fig. 6, A and B). We also compared the protein levels of phosphorylated OsMPK3 and OsMPK6 following the chitin and flg22 treatment. In the wild-type and STTM76113-A plants, rapid accumulation of phosphorylated OsMPK3 and OsMPK6 triggered by the PAMP treatment was observed; however, protein phosphorylation of OsMPK3 and OsMPK6 was attenuated in the *cngc5* mutant plants (Fig. 6, C and D). These results indicated that lsiR76113 participates in PTI responses by suppressing *OsCNGC5* expression. In contrast, the electron leakage as an indicator of hypersensitive cell death during ETI was no difference among NPB, STTM76113-A mutant, and *cngc5-1c* mutant plants (Supplemental Fig. S7), confirming that lsiR76113-mediated immunity involved in PTI response.

## Discussion

RNA silencing is a conserved mechanism of gene regulation at transcriptional and post-transcriptional levels. siRNAs with a length of 21 to 24 nt are known to play an extensive regulatory role in plant development and responses to biotic and abiotic stressors (Huang et al. 2019a). long siRNAs (lsiRNAs), a class of sRNA with a length of 25 to 40 nt, were identified in 2007. The lsiRNA, AtlsiRNA-1 was found to be involved in plant immunity against avirulent pathogen (Katiyar-Agarwal et al. 2007). Subsequently, 25 to 40 nt lsiRNAs in rice were identified, and their expression was shown to respond to the invasion of *R. solani* (Niu et al. 2018). Sharp start and end positions based on DeepSeq and homogeneous bands without trailing sequences based on northern blot detection distinguished lsiRNAs from spontaneous RNA degradation products (Niu et al. 2018). In this study, we analyzed the lengths of the detected sRNA. Unexpectedly, compared with siRNA with a length of 21 to 24 nt, the abundance of the lsiRNA with a length of 25 to 30 nt was substantially higher (Fig. 1A), indicating that lack of information on lsiRNAs may not be due to their low relative abundance but rather a lack of understanding of their function. We also showed that infection with *M*. *oryzae* altered the expression profile of Os-lsiRNA, such as inhibiting the expression of lsiR76113 (Fig. 1B), which is consistent with a previous report showing that the expression of Os-lsiRNA responded to infection by fungal pathogens (Niu et al. 2018). AtlsiRNA-1 is induced by the bacterial pathogen *Pst* (*avrRpt2*) and involved in plant immunity by silencing the negative regulator of plant disease resistance (Katiyar-Agarwal et al. 2007). In our study, we also found that plants enhance plant disease resistance by down-regulating lsiR76113 to release the inhibition of *OsCNGC5* (Fig. 2). These findings suggest that lsiRNA may be implicated in the regulation of plant disease resistance and indicate that the abundant 25 to 30 nt lsiRNA may be a potent resource for studying the regulatory mechanism of plant autoimmunity in response to various pathogens.

CNGCs can be divided into four categories based on their phylogenies. OsCNGC4-6 and AtCNGC5-9 are located in Group II and conserved in all terrestrial plants (Nawaz et al. 2014). Although research on Group II OsCNGCs is still in its infancy, their homologous proteins in Arabidopsis have been studied in depth. However, the localization of AtCNGC7 and AtCNGC8 remains controversial. Both YFP-AtCNGC7 and YFP-AtCNGC8 were shown to have similar localization patterns in the endomembrane compartment rather than the plasma membrane (Chang et al. 2007; Zhao et al. 2021). More recently, Tunc-Ozdemir reported strong fluorescent signals of GFP-AtCNGC7, which were predominantly associated with the plasma membrane (Tunc-Ozdemir et al. 2013). YFP-AtCNGC5 and YFP-AtCNGC6 localized to the periphery of *N. benthamiana* protoplasts and overlapped with the plasma membrane. Moreover, AtCNGC5 and AtCNGC6, which act as nonselective Ca^2+^-permeable cation channels, are required for cGMP-activated inward-conducting cation currents in Arabidopsis guard cells (Wang et al. 2013). The functions of AtCNGC5 and AtCNGC6 as calcium channels are consistent with the regulatory role of OsCNGC5 for cytosolic calcium concentrations (Fig. 4). OsCNGC4 shares the highest similarity to OsCNGC5 in rice. Whether OsCNGC4 mediates a similar process in immune responses must be studied, and the possible interactions between OsCNGC4 and OsCNGC5 require further exploration. The impaired disease resistance of *cngc5* mutant rice against blast disease could be rescued by a sufficient concentration of exogenous Ca^2+^ (Fig. 5 and Supplemental Fig. S6), suggesting the existence of other calcium channels with redundant functions that allow the transient increase in cytosolic Ca^2+^ to activate plant calcium-dependent immunity. This observation is consistent with a previous report that OsCNGC9 mediates PAMP-induced Ca^2+^ influx and calcium-dependent immune response (Wang et al. 2019).

CNGC expression has been reported to be affected by biological and abiotic stressors. Under cold stress conditions, the expression levels of 10 OsCNGCs belonging to phylogenetic Groups I, II, and III are significantly upregulated (Nawaz et al. 2014). A recent study has shown that the expression of OsCNGC14 and OsCNGC16 is also rapidly induced in response to chilling stress. OsCNGC14 and OsCNGC16 are both localized in the plasma membrane and required for heat and chilling tolerance (Cui et al. 2020). Nawaz et al. (2014) reported that all 14 identified OsCNGCs (except OsCNGC5 and 6) were upregulated upon treatment with the bacterial pathogen *P*. *fuscovaginae*. Indeed, our results suggested that the expression of *OsCNGC5* was up-regulated by challenge with pathogenic fungi and is involved in the immune response to biotic stress (Fig. 1).

Extracellular signals are transformed into intracellular signals via cation flow, which regulates the physiological activities of cells (Flynn et al. 2001). Among them, Ca^2+^ signal is a very important second messenger, which can participate in various life activities of plants, including flowering regulation, root hair development, stress response, plant immunity, plant–microbe symbiosis, etc. (Furuyama and Dzelzkalns 1999; Gleason et al. 2006; Zheng et al. 2013; Tan et al. 2020; Jiang and Ding 2023). For example, in the symbiosis of *Medicago* and rhizobia, plant cells will recognize the Nod factor of rhizobia and activate Ca^2+^ channels. Nod factors induce Ca influx leading to a Ca^2+^ spike, which is recognized by calbindin and directs downstream phosphorylation (Gleason et al. 2006; Wang et al. 2022). Overall, plant calcium signaling functions as a complex regulatory network that influences various biological processes. These interconnected processes collectively enable plants to adapt to changing environments, facilitating normal growth, development, and adaptive reactions.

Members of the Arabidopsis CNGC family are generally considered to be located and function in the plasma membrane. When the cell recognizes the external stimulus through the receptor protein located on the cell membrane, it activates adenylate cyclase in the cytoplasm and the generated cyclic nucleotide opens the CNGCs and causes extracellular Ca^2+^ to flow in. Consistent with the CNGCs in Arabidopsis, our results showed that the *cngc5* mutation led to a decrease in cytosolic Ca^2+^, whereas in the knock-down mutant of lsiRNA76113, which is a repressor of *OsCNGC5*, a higher level of cytosolic Ca^2+^ was observed (Fig. 4, A and B), suggesting that *OsCNGC5* plays an important role in Ca^2+^ influx in rice plants. The influx of Ca^2+^ binds to calmodulin-binding protein to activate NO synthase, and then the second messenger small molecule NO is produced and participates in the downstream HR or autoimmune cascade. Ca^2+^ in the cytoplasm can also activate Ca^2+^-dependent protein kinases (CDPKs) to phosphorylate downstream target proteins. In contrast, Ca^2+^ in the cytoplasm inhibits the activity of CNGCs by activating calmodulin, thereby inhibiting the influx of Ca^2+^ through CNGCs and preventing a continuous increase in intracellular Ca^2+^ concentration (Ali et al. 2007; Demidchik and Shabala 2018).

**Figure 7.**
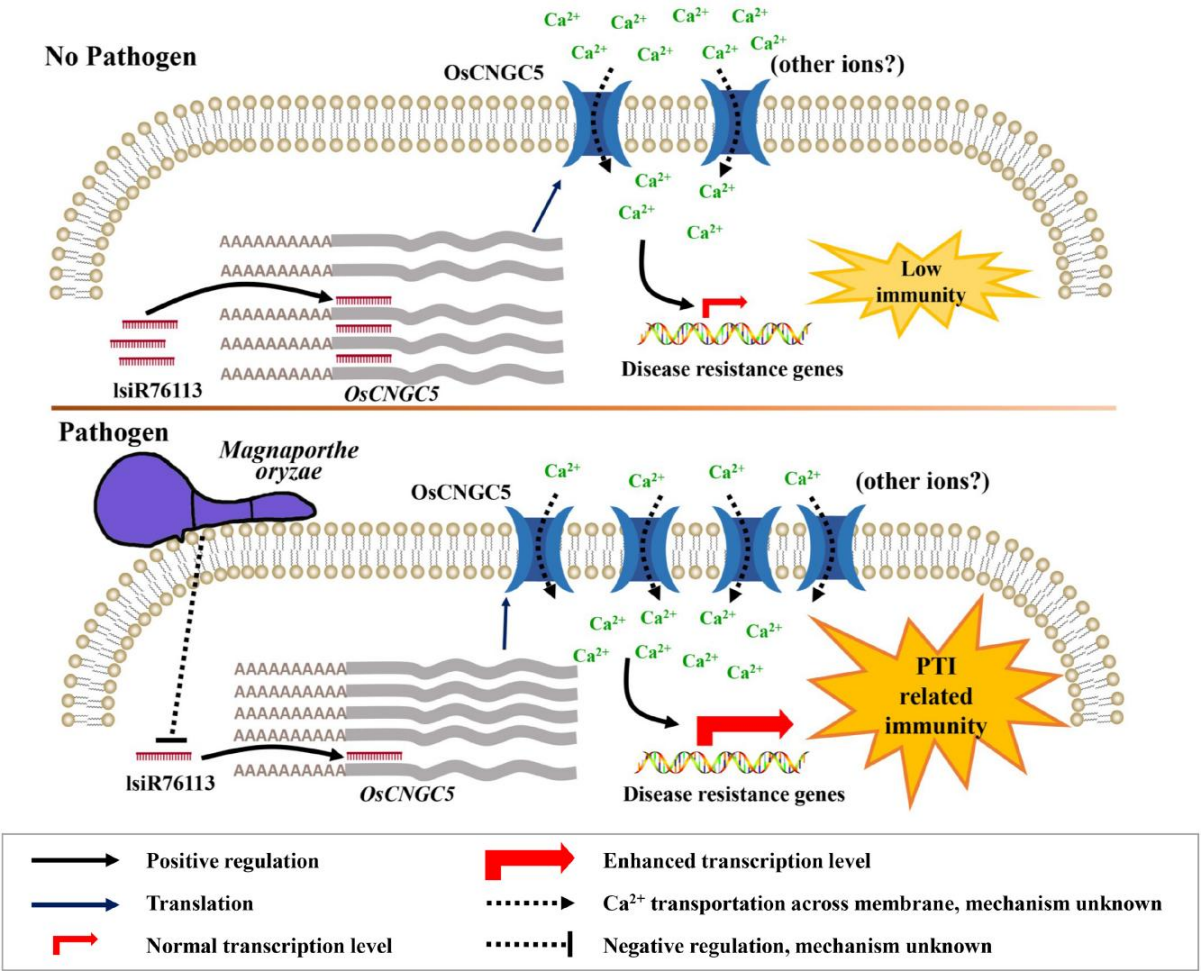
Proposed model for the regulatory role of lsiR76113 on the OsCNGC5 channel through the calcium-based PTI signaling pathway. Upper diagram: Before exposure to pathogen, plasma membranes maintain less OsCNGC5 channel to keep a low level of cytoplasmic Ca^2+^, resulting in inactivated disease resistance. Bottom diagram: Upon pathogen invasion, expression of lsiR76113 is suppressed, which derepresses the CNGC5 channel, triggering Ca^2+^ influx. The increased in cytosolic Ca^2+^ facilitates plant PTI responses and activates plant disease resistance. “other ions?”, The calcium ion specificity of CNGC5 is undetermined. PTI, pathogen-associated molecular pattern (PAMP)-triggered immunity.

A rapid increase in cytosolic Ca^2+^ is a hallmark of PTI (Yuan et al. 2017). Ca^2+^ influx is also required for hypersensitive cell death during ETI (Moeder et al. 2019). Several CNGCs have been identified to be involved in PTI and/or ETI by controlling the transient cytosolic Ca^2+^ influx. CNGC2 and CNGC4 participate in PTI and ETI responses. The *cngc2* and *cngc4* null mutants (*dnd1* and *dnd2*) showed impaired HR cell death caused by avirulent pathogens, indicating that Ca^2+^-dependent signaling mediated by CNGC2 and CNGC4 is required for ETI (Clough et al. 2000; Jurkowski et al. 2004). In contrast, the activation of the calcium channel formed by CNGC2 and CNGC4 relies on BIK1 phosphorylation upon pathogen treatment, thus linking this calcium channel to the PTI signaling pathway (Tian et al. 2019). Moreover, the *cngc20*-*4* gain-of-function mutation in *CNGC20* led to ectopic Ca^2+^ influx and enhanced both PTI responses and ETI-hypersensitive cell death (Zhao et al. 2021). AtCNGC11 and 12 show positive regulatory roles in *R* gene-mediated resistance responses against avirulent oomycetes and bacterial pathogens (Moeder et al. 2011). In rice, OsCNGC9 was shown to mediate PAMP-induced Ca^2+^ influx, which is critical for PAMP-triggered ROS burst, induces the expression of PTI-related defense genes, and positively regulates rice resistance to blast disease (Wang et al. 2019). Similar to OsCNGC9, our results showed that OsCNGC5 participates in the PTI response by mediating flg22-triggered ROS burst by activating Ca^2+^ influx (Fig. 4). The induction of PTI-related defense gene expression and MAPK phosphorylation further demonstrated the role of OsCNGC5 in PTI (Fig. 6). Overall, the recognition of M. oryzae infection leads to a decrease in the expression level of lsiR76113 and an increase in the expression level of its target gene OsCNGC5. Plant cells have more OsCNGC5 channel proteins that can induce more powerful Ca^2+^ spikes in immune responses, eliciting a stronger immune response (Fig. 7). Currently, there are more and more studies on the involvement of CNGCs proteins in plant disease resistance. People are also interested in how to use CNGCs proteins to enhance plant disease resistance. There will be more related discoveries in the future.

## Materials and methods

### Plant growth conditions and pathogen infection

Rice (*O. sativa*) NPB was used as the wild-type line; the STTM76113 and *cngc5* mutant lines were generated from an NPB background; and *Nicotiana benthamiana* was used for transient protein expression. The plants were grown in a greenhouse at 28°C and 70% relative humidity under 12-h/12-h light/dark cycles.

*Magnaporthe oryzae* strain Guy11 and the eGFP-tagged Zhong1 strain were chosen as pathogens. *Magnaporthe oryzae* was incubated in complete medium (CM) at 28°C for 7 days and transferred to RDC medium. The fungus was then incubated under UV black light at 28°C for approximately 5 days for sporulation. The spores were collected and adjusted to 1 × 10^5^ spores mL^−1^ for inoculation. The rice blast resistance assay was performed as previously described (Wang et al. 2018).

*Magnaporthe oryzae* strain Guy11 was used for spray or punch inoculation. For spray inoculation, three-leaf-stage plants were spray-inoculated with gelatin or the indicated spore suspensions (1 × 10^5^ spores mL^−1^ in 0.2% w/v gelatin solution). The inoculated plants were kept in the dark at 80% relative humidity for 24 h before being transferred to a growth chamber under the following conditions: 25°C, 80% relative humidity, and a 12-h/12-h light/dark photoperiod. For punch inoculation, the isolated 1-month-old leaves were slightly wounded with a pin-punch and kept in moist petri dishes with 1.5 mm Ca^2+^ or without Ca^2+^ supplementation. Ten microliters of spore suspension (1 × 10^5^ spores mL^−1^) was added to the wound for pathogen inoculation. Five (normal) or seven (exogenous Ca^2+^ application) days later, the lesion size was analyzed using ImageJ software. Measurement of DNA amount of *M. oryzae Pot2* against the rice genomic ubiquitin DNA by qPCR was used for relative fungal biomass calculation. To observe the infection process of *M. oryzae*, 5-cm-long isolated leaf sheaths were injection-inoculated with spore suspension. The epidermal layer was carefully excised using a razor blade. Fluorescence was visualized by fluorescence microscope (Zeiss Axio Observer 3) at 24 and 36 hpi.

For *Rhizoctonia solani* inoculation, the fungus (AG1-IA) was grown on PDA medium at 28°C for 48 h. Actively growing *R*. *solani* mycelium plugs (5 mm in diameter) were inoculated on 2-week-old isolated leaves and incubated for 48 h at 28 °C and a 12-h/12-h light/dark photoperiod. Photographs were taken with a digital camera and the size of the lesions was analyzed with ImageJ software. Relative fungal biomass was calculated by measuring DNA amount of *R*. *solani Actin* against the rice genomic ubiquitin DNA by qPCR.

### sRNA library construction

A least 20 rice spray-inoculated seedlings from the same treatment group were pooled for RNA extraction, library construction, and RT-qPCR analysis. Plants that received the same treatment and were maintained under the same conditions were used as biological replicates. sRNA library construction and Illumina sequencing were performed as previously described (Mi et al. 2008). sRNA reads with a length of over 16 nucleotides were mapped to the rice nuclear, chloroplast, and mitochondrial genomes (http://rice.plantbiology.msu.edu/; version 6.0) and *M. oryzae* genomes (http://www.broadinstitute.org). Perfect genome-matched sRNAs were analyzed as previously described (Wu et al. 2009). The normalized abundance of the sRNAs was calculated as the number of reads per million.

### Total RNA and genomic DNA extraction

Total RNA was extracted from rice leaves or rice protoplasts using Trizol reagent (Invitrogen) and used for gene expression testing. Genomic DNA was extracted using the CTAB method, as described previously (Aboul-Maaty and Oraby 2019). Genomic DNA was used for the *M. oryzae* biomass measurement and target gene sequencing. A NanoDrop One system (Thermo Fisher Scientific) was used to detect the quality and quantity of RNA or DNA.

### Northern blot analysis

RNA blot analyses of miRNAs from the total extracts were performed as described previously (Katiyar-Agarwal and jin 2007). Inoculated plants were used for RNA extraction at 0, 24, 48, and 120 hpi. Total RNA was extracted using Trizol reagent (Invitrogen) according to the manufacturer's instructions. RNA was resolved on a 14% denaturing 8 m urea-PAGE gel, transferred, and chemically cross-linked onto a Hybond N^+^ membrane (GE Healthcare Life Sciences) using N-(3-dimethylaminopropyl)-N9-ethylcarbodiimide hydrochloride. The miRNA probes (Supplemental Table S2) were end-labeled with [γ-^32^P] ATP using T4 polynucleotide kinase (New England Biolabs). The expression levels were quantified using ImageJ software.

### RT-qPCR and qPCR

RNA was reverse-transcribed into cDNA using the PrimeScript RT Reagent Kit (Takara). RT-qPCR was performed using the AceQ qPCR SYBR Green Master Mix (Low ROX Premixed) (Vazyme). The transcript levels of each gene were measured using an Applied Biosystems 7500 system according to the manufacturer's instructions. 18 s rRNA was used as a quantitative control in the RT-qPCR analysis. The primers used in this study are listed in Supplemental Table S2. For biomass qPCR test, *MoPot2* primer, *XOO*114 primer and *R. solani* Actin primer were used for *M. oryzae*, *Xoo*, and *R. solani*, respectively. *OsUbq* was chose as quantitative control. For gene expression level test, Os18S rRNA primer was chose as quantitative control. For sRNA expression level test, lsiR76113 stem-loop primer were used for reverse transcription. lsiR76113-QF and Universal primer-R were used for lsiR76113 expression level test. Rice-U6 primer was chose as quantitative control.

### Transient expression analysis in *Nicotiana benthamiana*

The sequence of lsiR76113 or lsiR118113 was cloned into the pRS300 vector to generate the lsiR76113/lsiR118113 expression module with primers OE-RS300-76113-(I-IV) or OE-RS300-118113-(I- IV) (http://wmd3.weigelworld.org). The module was cloned into pBIN-3HA by EcoRI (Takara) and BamHI (Takara) sites to express lsiR76113 in plants. YFP-wtOsCNGC5-3′-UTR or YFP-muOsCNGC5-3′-UTR fragments (constructed by polymerase chain reaction with YFP fragment, wt/muCNGC5-3′UTR fragment, TY-CNGC5-3′UTR-F/R, TY-muCNGC5-3′UTR-F/R, and TY-YFP-F primers) were cloned into the pBIN-3FLAG vector to generate overexpression vectors by homologous recombination (Vazyme Mut Express II Fast Mutagenesis Kit V2) with TY-RS300-miRNA OE-F/R primers. Transient coexpression assays were performed by infiltrating 3-week-old *N*. *benthamiana* plants with *A*. *tumefaciens* GV3101 (OD600 = 1.0) harboring constructs containing lsiR76113 (pBIN-3HA) or *A*. *tumefaciens* GV3101 (OD600 = 1.0) containing YFP-wtOsCNGC5-3′-UTR or YFP-muOsCNGC5-3′-UTR (pBIN-3FLAG). Leaf tissues were collected 48 h after infiltration. GFP fluorescence of *N*. *benthamiana* leaves were visualized with excitation at 470 nm and emission at 500 to 550 nm by VILBER Fusion FX7(Spectra) Plant Live Imaging System (Fig. 2B) or Leica TCS SP8 confocal microscope (Fig. 2D) after 48 h post-infiltration. GFP fluorescence was visualized with GFP excitation at 488 nm and emission at 495 to 548 nm. The detector type is PMT. Gain value for the detector was set to 783 V.

### Western blot

The tissue was ground in liquid nitrogen, and total protein was extracted using 2×SDS loading buffer. The samples were resolved on a 12% SDS-PAGE gel and transferred onto an Amersham Hybond-P PVDF membrane (GE Healthcare) using Tris-Gly transfer buffer. Membranes were blocked with 5% (w/v) milk in 0.05% (v/v) TBS-plus Tween 20 (TBST) for 40 min and incubated overnight at 4°C with 1:5,000 dilutions of primary antibodies of mouse anti-Flag conjugated to horseradish peroxidase (Abmart), washed three times with TBST, and incubated overnight at 4°C with 1:1,000 dilutions of secondary antibodies (Beyotime). The membranes were then washed three times with TBST. Detection was performed using ECL Plus western blotting Detection Reagents (GE Healthcare) and ChemiDoc Touch Imager (Bio-Rad). Anti-GFP antibody (Abmart) was used to determine the expression level of the YFP protein. Ponceau was used as a loading control, and MAPK assays were performed as previously described (Zhang et al. 2021). Briefly, 1 mm of flg22 peptide was infiltrated into the leaves of 4-week-old plants. Total protein samples were collected at 0, 5, 10, 20, and 30 min and used for immunoblotting with an anti-p44/42 mAPK antibody (Cell Signaling Technology) to detect activated forms of MPKs. Image data were analyzed using Image Lab Software (Bio-Rad) and assembled using Adobe Photoshop CS6.

### Generation of transgenic plants

The *cngc5* mutants were constructed using CRISPR technology (Lei et al. 2014). The selection of highly specific targets and construction plans is described at http://crispr.hzau.edu.cn/. Briefly, the chosen target regions in the *OsCNGC5* coding sequence (CDS) were confused with OsU6a/b (part of pYLgRNA-OsU6a/b). Then, the fragment was cloned into the pYLCRISPR/Cas9-MB vector with the help of BsaI restriction enzyme (New England Biolabs) and 2 × Phanta Max Master Mix (Vazyme) high-fidelity DNA polymerase. STTM mutants were constructed according to a previously described method (Tang et al. 2012). Briefly, primers (lsiR76113-STTM Swa48ntlink-PF/PR) harbor short tandem target mimic (STTM) sequence of lsiR76113 were confused with pOT2 fragment by polymerase chain reaction. Then, the fragment was digested with swaI restriction enzyme (New England Biolabs). After that, the fragment was self-ligated with the help of T4 DNA Ligase (Takara) to generate pOT2-STTM76113 vector. After verification by sequencing, pOT2-STTM76113-PacI fragment was clone from pOT2-STTM76113 vector with Origin-del-PacI-PF/PR primers. Than the pOT2-STTM76113-PacI fragment and pFGC5941 vector digested with PacI restriction enzyme (Takara) were ligated with each other to get destination vector with the help of T4 DNA Ligase (Takara). The constructs were transferred to *Agrobacterium* strain EHA105 for the generation of transgenic rice lines by Wuhan Boyuan Biotechnology Co. Ltd. *cngc5* mutants were verified by DNA sequencing of the target sites. STTM mutants were verified by measuring RNA expression levels using stem-loop RT-qPCR (Chen et al. 2005). Primers used for transgenic plant verification are listed in Supplemental Table S2.

### Cytoplasmic Ca^2+^ measurement

Rice protoplasts were isolated and transfected as previously described (Zhang et al. 2011). Plasmids containing GCaMP3, a reporter for visualizing cytosolic Ca^2+^, were transfected into protoplasts isolated from the leaves of 16-day-old plants as described previously (Zhang et al. 2011; Zhao et al. 2021). After 16 h incubation in 25°C, GCaMP3 fluorescence was visualized with GFP excitation at 488 nm and emission at 491 to 657 nm using a ZEISS LSM 980 with an Airyscan2 confocal microscope. Detector type is GaAsP-PMT. Gain value for the detector was set to 700 V. Fluorescence data of the images were calculated using ImageJ software.

### DAB staining assay

Plant tissues were stained with DAB to determine the accumulation of H_2_O_2_, as described previously (Wang et al. 2018). Entire plants of three-leaf-stage seedlings were inoculated by spray inoculation with 1 × 10^5^ spores mL^−1^ Guy11 spores and incubated in the dark at 25°C for 36 h. Leaf sections were collected and placed in 1 mg mL^−1^ DAB (Sigma) and shaken gently in the dark at room temperature for 8 h. The leaf sections were then decolorized in an ethanol:acetic acid (94:4) solution at room temperature for 8 h in the dark. The leaves were rinsed with water to remove reagent residues. The stained leaves were observed under an Olympus/IX71 microscope.

### Ion leakage assay

Cell death was assayed by measuring the ion leakage from leaf discs, as previously described (Wang et al. 2018). Briefly, 10 leaf discs (0.5 cm diameter) of each sample were immersed in distilled water for 3 h at room temperature. Then, the conductivity of the bathing solution was measured using a conductivity meter (Bante 950) to obtain the initial conductivity value A. The leaf discs were then transferred to the bathing solution and boiled for 25 min in sealed tubes. The total conductivity value (B) was measured after cooling the solution to room temperature. The ion leakage rate was calculated as the ratio of the initial to the total conductivity (A/B × 100%).

### ROS burst measurement

ROS bursts were detected as previously described (Wang et al. 2019). Briefly, round leaf discs (0.5 cm) were excised from 2-month-old plants, followed by overnight incubation in a 96-well plate with 200 *µ*L H_2_O with 1.5 mm Ca^2+^ or without Ca^2+^. H_2_O was replaced by 200 *µ*L of reaction solution (20 *µ*M luminol, 1 *µ*g mL^−1^ of horseradish peroxidase) supplemented with 500 nm flg22. ROS measurements were conducted immediately using a PE/EnSight System with a 1-min interval reading time over a period of 30 min.

### Measurements of net Ca^2+^ flux

Net Ca^2+^ flux was measured by Non-invasive Micro-test Technique (NMT-YG-150, USA) as previously described with minor modifications (Wang et al. 2019). Root tip of 1-week-old rice seedling were washed gently with measuring buffer (0.2 mm CaCl_2_, 0.1 mm NaCl, 0.1 mm MgCl_2_ and 0.1 mm KCl, pH 5.2) for 30 min equilibration. Prior to PAMPs treatment, steady-state flux in leaf mesophyll cells was continuously recorded for 2 min. Chitin (Santa Cruz Biotechnology) were slowly added to the measuring buffer until the chitin concentration reached 10 *μ*M. Subsequently, the transient flux of Ca^2+^ was recorded for 5 min.

### FM4-64 staining

*Nicotiana benthamiana* leaves were stained with FM4-64 (MedChem Express) as described (Tian et al. 2015). Briefly, CNGC5 were confused with YPF fragment with primers for CNGC5 sub-localization experiment in Supplemental Table S2. After that, CNGC5-YFP fragment were clone into pNC-Cam1304-35S vector by Nimble Cloning Mix (NC Biotech). *Nicotiana benthamiana* leaves agroinfiltrated with pNC-Cam1304-35S vector or the vector harbors CNGC5-YFP fragment for 48 h were cut in small fragments, incubated in a medium containing 5 *μ*g mL^−1^ FM4-64 for 40 min. Leaf fragments were rinsed several times with water and observed under Leica TCS SP8 confocal microscope. GFP fluorescence was visualized with GFP excitation at 488 nm and emission at 492 to 549 nm. Detector type is PMT. Gain value for the detector was set to 869.4 V. RFP fluorescence was visualized with excitation at 488 nm and emission at 573 to 640 nm. Detector type is HyD. Gain value for the detector was set to 155.3 V. Other setup: Argon output power was 14.29% on, Laser lines intensity was 28.65%.

### Accession numbers

Sequence data from this study can be found in the Rice Genome Database (http://rice.plantbiology.msu.edu/), The Rice Annotation Project (RAP) (https://rapdb.dna.affrc.go.jp/) under the following accession numbers: *OsGNGC5* (*LOC_Os12g28260*), *OsKS4* (*LOC_Os04g10060*), and *OsPAL1* (*LOC_Os02g41630*).

## Supplementary Material

kiad599_Supplementary_Data

## Supplemental data

The following materials are available in the online version of this article.

**Supplemental Figure S1.** Length distribution of sequenced sRNA.

**Supplemental Figure S2.** Transgenic plants validation of lsiR76113 knock-down and *cngc5-1c* mutant.

**Supplemental Figure S3.** lsiR76113 silences the expression level of *OsCNGC5* but not *OsMyosin*.

**Supplemental Figure S4.** lsiR76113 promoted rice resistance against rice sheath blight and bacterial blight.

**Supplemental Figure S5.** Plasmids containing GCaMP3 can be used as a reporter for visualizing cytosolic Ca^2+^.

**Supplemental Figure S6.** Effect of gradient external Ca^2+^ on disease resistance.

**Supplemental Figure S7.** OsCNGC5 does not affect rice ETI response.

**Supplemental Table S1.** List of identified lsiRNA differentially expressed in *M. oryzae* infected rice.

**Supplemental Table S2.** Primer information.

**Supplemental Table S3.** Vectors used in the study.

## Dive Curated Terms

The following phenotypic, genotypic, and functional terms are of significance to the work described in this paper:

AtCNGC11 Gramene: AT2G46440
AtCNGC11 Araport: AT2G46440

AtCNGC12 Gramene: AT2G46450
AtCNGC12 Araport: AT2G46450

ETI Gramene: Effector-triggered immunity
ETI Araport: Effector-triggered immunity

PTI Gramene: pattern-triggered immunity
PTI Araport: pattern-triggered immunity

ATP CHEBI: CHEBI:15422

PAL1 Gramene: Os02g41630
PAL1 Araport: Os02g41630

chitin CHEBI: CHEBI:17029

KS4 Gramene: Os04g10060
KS4 Araport: Os04g10060

OsCNGC5 Gramene: Os12g28260
OsCNGC5 Araport: Os12g28260

OsCNGC9 Gramene: Os09g38580
OsCNGC9 Araport: Os09g38580

OsCNGC14 Gramene: Os03g55100
OsCNGC14 Araport: Os03g55100

OsCNGC16 Gramene: Os05g42250
OsCNGC16 Araport: Os05g42250

DND1 Gramene: AT5G15410
DND1 Araport: AT5G15410

AtCNGC19 Gramene: AT3G17690
AtCNGC19 Araport: AT3G17690

OsCNGC4 Gramene: Os03g4444
OsCNGC4 Araport: Os03g4444

AtCNGC2 Gramene: AT5G15410
AtCNGC2 Araport: AT5G15410

AtCNGC4 Gramene: AT5G54250
AtCNGC4 Araport: AT5G54250

AtCNGC5 Gramene: AT5G57940
AtCNGC5 Araport: AT5G57940

AtCNGC6 Gramene: AT2G23980
AtCNGC6 Araport: AT2G23980

AtCNGC7 Gramene: AT1G15990
AtCNGC7 Araport: AT1G15990

AtCNGC8 Gramene: AT1G19780
AtCNGC8 Araport: AT1G19780

AtCNGC14 Gramene: AT2G24610
AtCNGC14 Araport: AT2G24610

AtCNGC17 Gramene: AT4G30360
AtCNGC17 Araport: AT4G30360

AtCNGC18 Gramene: AT5G14870
AtCNGC18 Araport: AT5G14870
