# Supplementary material for: Long small RNA76113 targets CYCLIC NUCLEOTIDE-GATED ION CHANNEL 5 to repress disease resistance in rice
Source: Plant Physiol. 2023 Nov 9;194(3):1889–905. doi: 10.1093/plphys/kiad599 (PMC10904327; doi:10.1093/plphys/kiad599)
Supplement: kiad599_Supplementary_Data [file kiad599_supplementary_data.zip › PP2023RA01587D____WORKON____Supplemental_Figures_4.pdf]

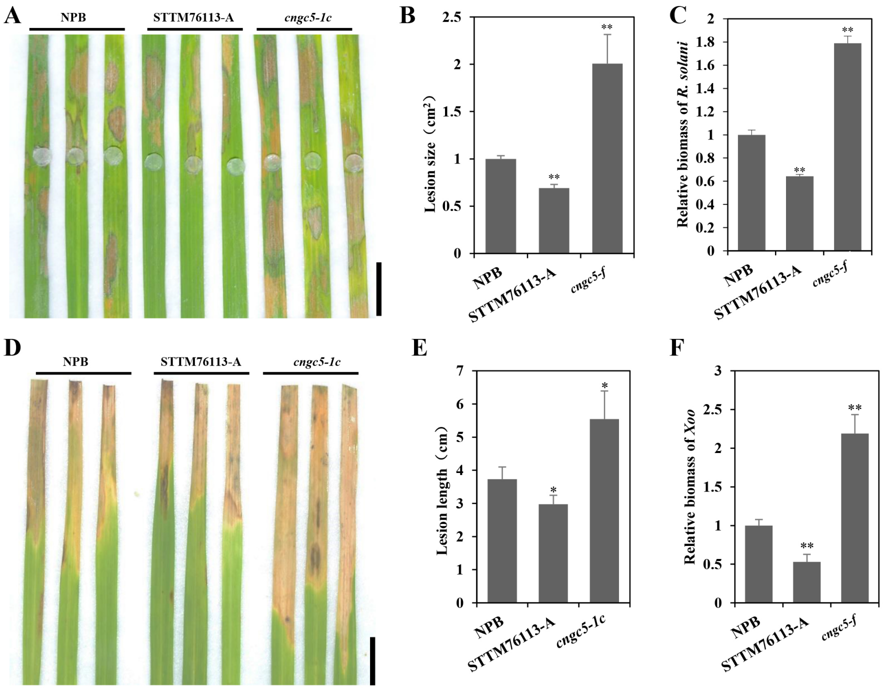

Supplemental Figure S4. lsiR76113 promoted rice resistance against rice sheath blight and bacterial blight. (A) Phenotypes of sheath blight disease in different lines. Scale bars, 1 cm. Lesion size (B) and relative biomass of *R. Solani* (C) of picture (A). (D) Phenotypes of rice bacterial blight disease in different lines. Scale bars, 1 cm. Lesion length (E) and relative biomass of *Xoo*. (F) of picture (D). Values are means ± SD (B/C/E/F, n= 3 replicates). The Student's t-test analysis indicates a significant difference (*P < 0.05, **P < 0.01).
