# Supplementary material for: Long small RNA76113 targets CYCLIC NUCLEOTIDE-GATED ION CHANNEL 5 to repress disease resistance in rice
Source: Plant Physiol. 2023 Nov 9;194(3):1889–905. doi: 10.1093/plphys/kiad599 (PMC10904327; doi:10.1093/plphys/kiad599)
Supplement: kiad599_Supplementary_Data [file kiad599_supplementary_data.zip › sup figure S17.pdf]

A

Class	counts	(%)
Total	11926	100
Intergenic	508	4.26
Promoter	411	3.45
miRNA	14	0.12
TE_and_repeat	10351	86.8
Gene_sense	606	5.08
Gene_antisense	36	0.3

B

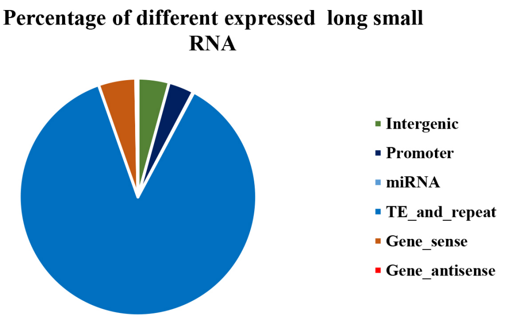

Supplemental Figure S1. Length distribution of sequenced sRNA. (A-B) The difference in lsiRNA expression levels between a *M. oryzae* spraying treatment and the control treatment (water) at 24 hours post inoculation were compared and analyzed. The reads obtained by sequencing mapped to different sources of the rice genome. TE, transposable elements.

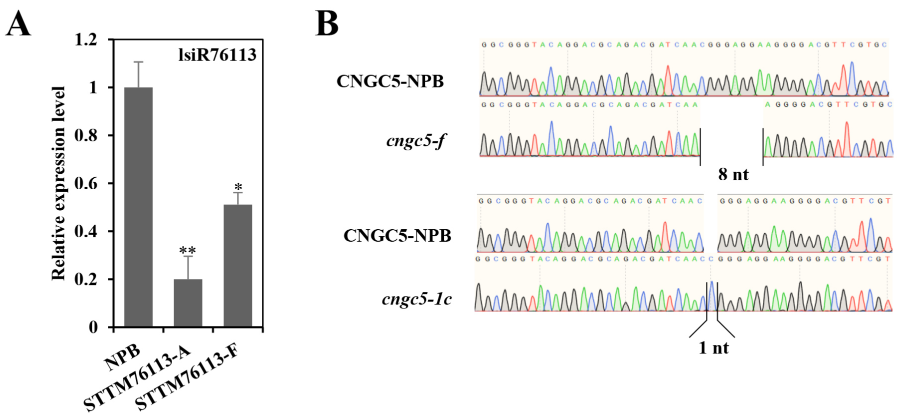

Supplemental Figure S2. Transgenic plants validation of lsiR76113 knock-down and *cngc5-1c* mutant. (A) Relative expression level of lsiR76113 in the STTM76113-A and STTM76113-F mutant lines (lsiR76113 knock-down mutants, short tandem target mimic (STTM) technology to block the functions of lsiR76113). NPB, *Oryza sativa* subsp. *Japonica*. The values are presented as means ± SD (A, n= 3 samples). The Student's t-test analysis indicates a significant difference (*P < 0.05, **P < 0.01). Three independent biological experiments were carried out, and all three repetitions showed similar results. (B) Alignment of target gene sequences in *cngc5-f* and *cngc5-1c* transgenic rice lines with wild type.

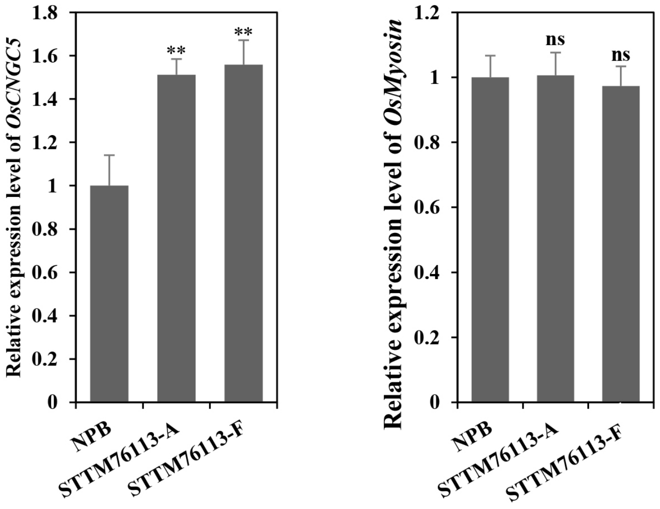

Supplemental Figure S3. lsiR76113 silences the expression level of *OsCNGC5* but not *OsMyosin*. The values are presented as means ± SD (n= 3 replicates). The Student's t-test analysis indicates a significant difference (*P < 0.05, **P < 0.01). ns, Not Significant. Three independent biological experiments were carried out, and all three repetitions showed similar results. NPB, *Oryza sativa* subsp. *Japonica*. STTM76113-A/F, (lsiR76113 knock-down mutants, short tandem target mimic (STTM) technology to block the functions of lsiR76113).

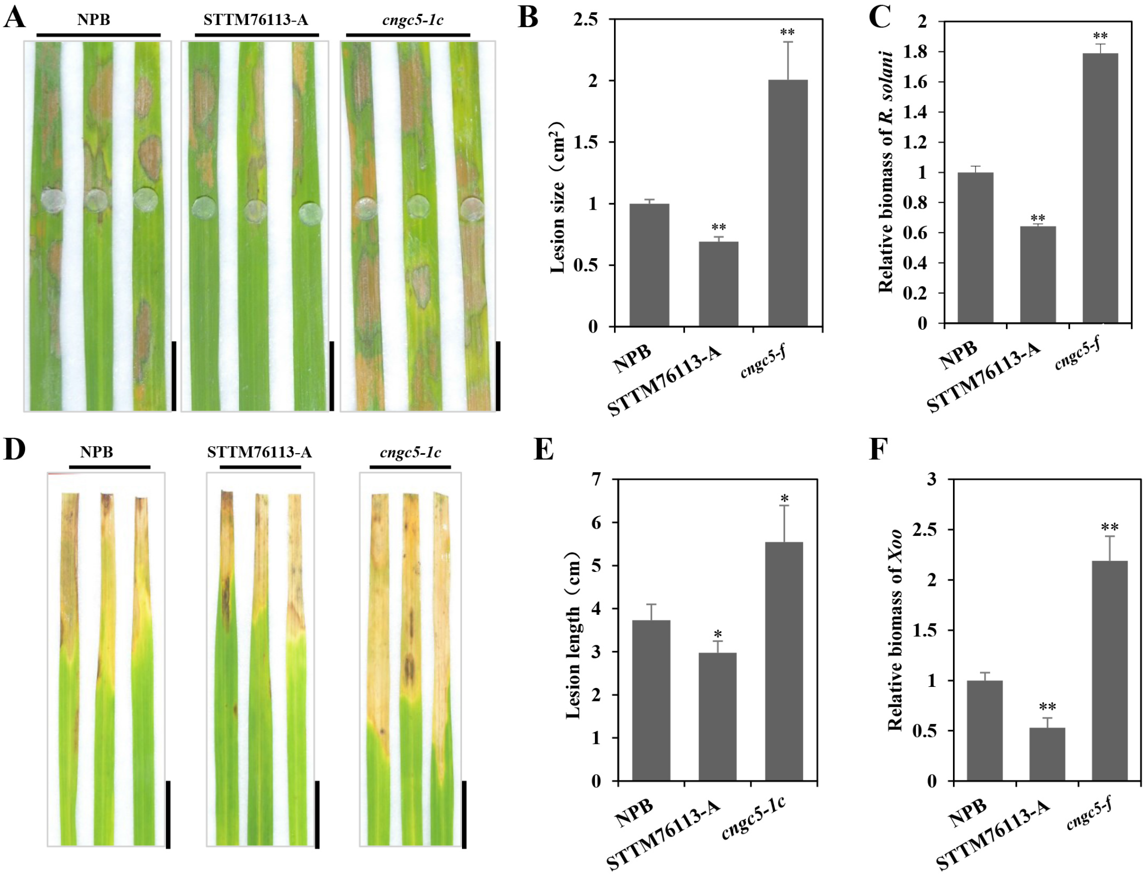

Supplemental Figure S4. lsiR76113 promoted rice resistance against rice sheath blight and bacterial blight. (A) Phenotypes of sheath blight disease in different lines. Scale bars, 1 cm. Lesion size (B) and relative biomass of *Rhizoctonia Solani* (C) of picture (A). (D) Phenotypes of rice bacterial blight disease in different lines. Scale bars, 1 cm. Lesion length (E) and relative biomass of *Xanthomonas oryzae* pv. *oryzae* (*Xoo*). (F) of leaves in picture (D). NPB, *Oryza sativa* subsp. *Japonica*. STTM76113-A, (lsiR76113 knock-down mutants, short tandem target mimic (STTM) technology to block the functions of lsiR76113). The values are presented as means ± SD (B/C/E/F, n= 3 replicates). The Student's t-test analysis indicates a significant difference (*P < 0.05, **P < 0.01). Two independent biological experiments were carried out, and both two repetitions showed similar results.

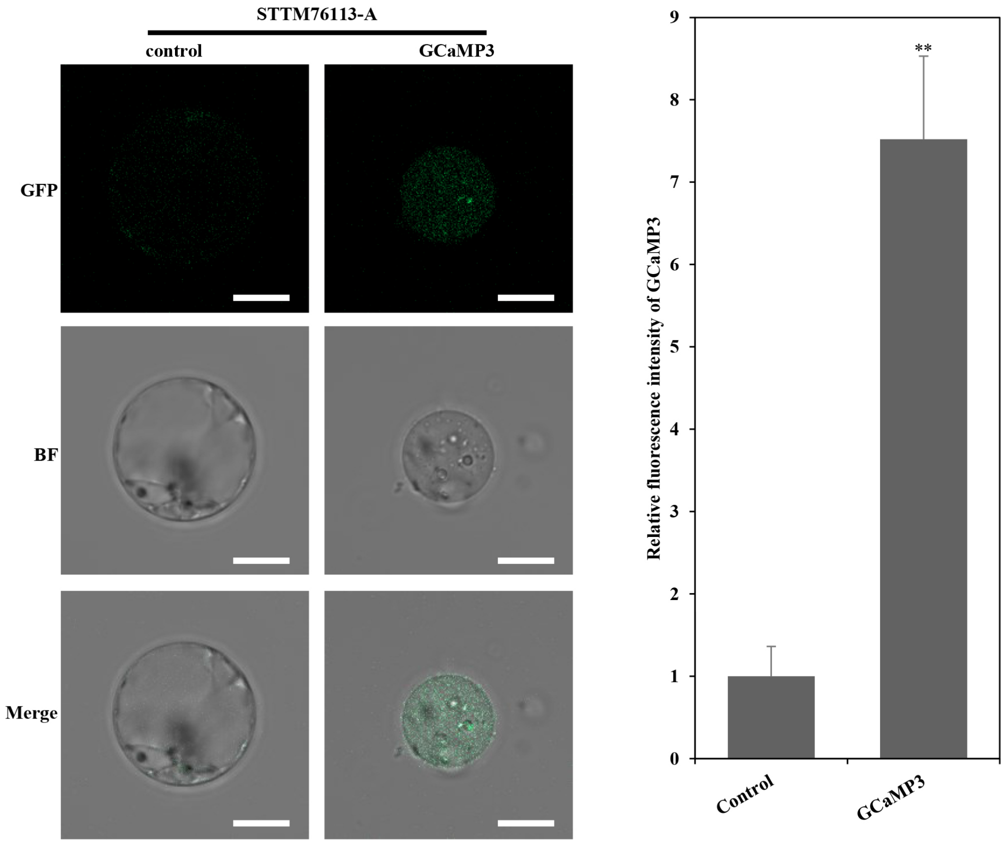

Supplemental Figure S5. Plasmids containing GCaMP3 can be used as a reporter for visualizing cytosolic Ca^{2+}. Scale bars, 10 µm. STTM76113-A, (lsiR76113 knock-down mutants, short tandem target mimic (STTM) technology to block the functions of lsiR76113). The values are presented as means ± SD (n= 3 replicates). The Student's t-test analysis indicates a significant difference (*P < 0.05, **P < 0.01). One biological experiment was carried out.

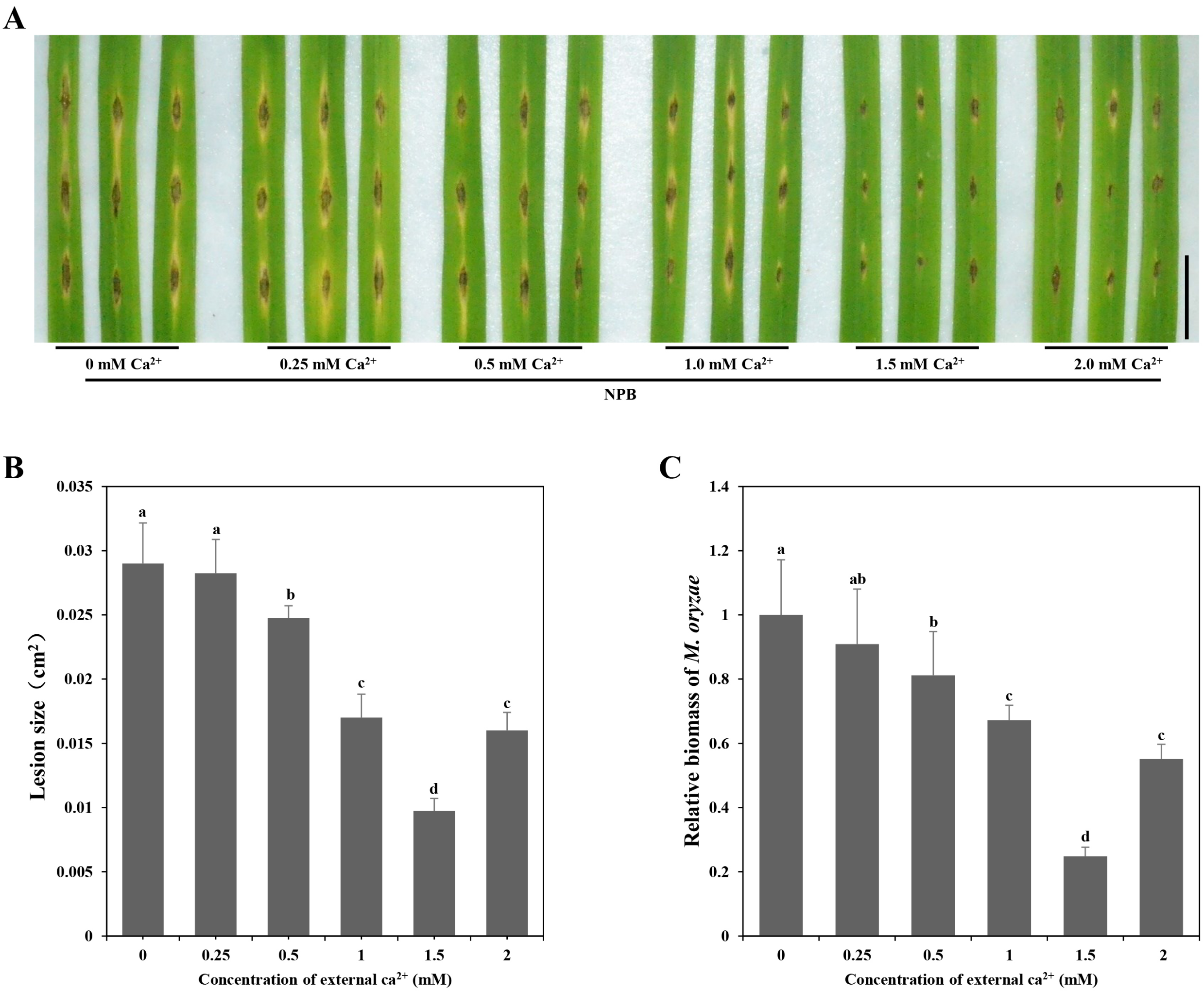

Supplemental Figure S6. Effect of gradient external Ca^{2+} on disease resistance. (A) Phenotype of rice blast disease after adding different concentrations of Ca^{2+}. NPB, *Oryza sativa* subsp. *Japonica*. Scale bar, 1 cm (B) Lesion size (B) and relative biomass of *M. oryzae* (C) of picture (A). The values are presented as means ± SD (B and C, n= 3 replicates). The Student's t-test analysis indicates a significant difference. Lowercase letter (a/b/c/d) indicates the significance level $\alpha = 0.05$. Those with the same letters indicate that the difference is not significant. Two independent biological experiments were carried out, and both two repetitions showed similar results.

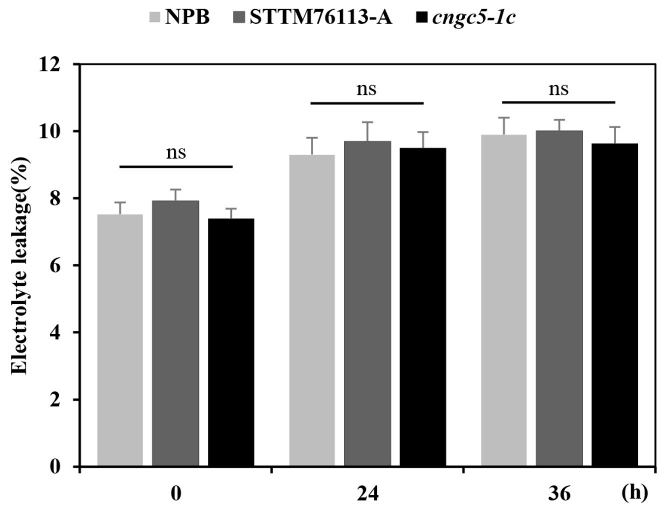

Supplemental Figure S7. OsCNGC5 does not affect rice ETI response. NPB, *Oryza sativa* subsp. *Japonica*. STTM76113-A, (lsiR76113 knock-down mutants, short tandem target mimic (STTM) technology to block the functions of lsiR76113). The values are presented as means ± SD (n= 3 samples). The Student's t-test analysis indicates a significant difference (*P < 0.05, **P < 0.01). Three independent biological experiments were carried out, and all three repetitions showed similar results. ns, Not Significant.
