# Supplementary material for: Long small RNA76113 targets CYCLIC NUCLEOTIDE-GATED ION CHANNEL 5 to repress disease resistance in rice
Source: Plant Physiol. 2023 Nov 9;194(3):1889–905. doi: 10.1093/plphys/kiad599 (PMC10904327; doi:10.1093/plphys/kiad599)
Supplement: kiad599_Supplementary_Data [file kiad599_supplementary_data.zip › Supplemental Figure S1.pdf]

A

Class	counts	(%)
Total	11926	100
Intergenic	508	4.26
Promoter	411	3.45
miRNA	14	0.12
TE_and_repeat	10351	86.8
Gene_sense	606	5.08
Gene_antisense	36	0.3

B

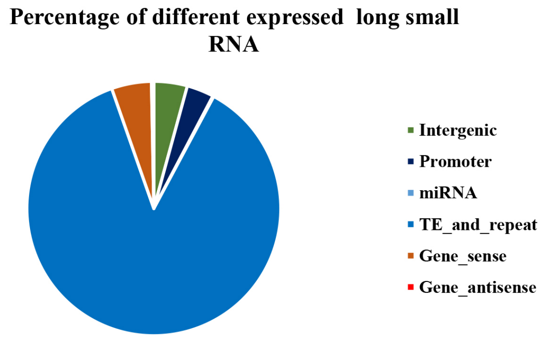

Supplemental Figure S1. Length distribution of sequenced sRNA. (A-B) The difference in lsiRNA expression levels between a *M. oryzae* spraying treatment and the control treatment (water) at 24 hours post inoculation were compared and analyzed. The reads obtained by sequencing mapped to different sources of the rice genome. TE, transposable elements.
