# Supplementary material for: Long small RNA76113 targets CYCLIC NUCLEOTIDE-GATED ION CHANNEL 5 to repress disease resistance in rice
Source: Plant Physiol. 2023 Nov 9;194(3):1889–905. doi: 10.1093/plphys/kiad599 (PMC10904327; doi:10.1093/plphys/kiad599)
Supplement: kiad599_Supplementary_Data [file kiad599_supplementary_data.zip › Supplemental Figure S2.pdf]

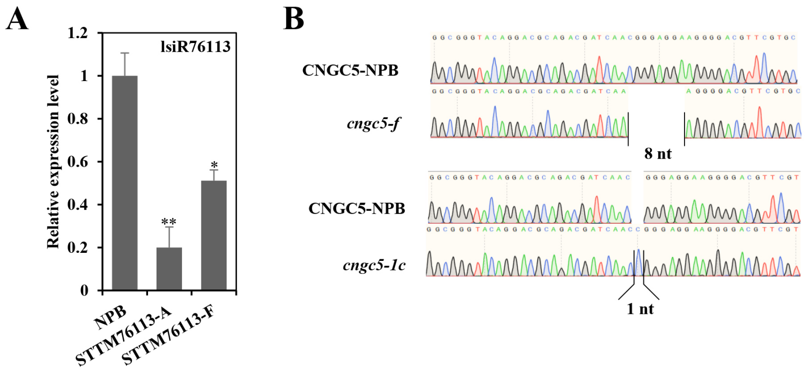

Supplemental Figure S2. Transgenic plants validation of lsiR76113 knock-down and *cngc5-1c* mutant. (A) Relative expression level of lsiR76113 in the STTM76113-A and STTM76113-F mutant lines. (B) Alignment of target gene sequences in *cngc5-f* and *cngc5-1c* transgenic rice lines with wild type. Values are means ± SD (A, n= 3 samples). The Student's t-test analysis indicates a significant difference (*P < 0.05, **P < 0.01).
