# Supplementary material for: Long small RNA76113 targets CYCLIC NUCLEOTIDE-GATED ION CHANNEL 5 to repress disease resistance in rice
Source: Plant Physiol. 2023 Nov 9;194(3):1889–905. doi: 10.1093/plphys/kiad599 (PMC10904327; doi:10.1093/plphys/kiad599)
Supplement: kiad599_Supplementary_Data [file kiad599_supplementary_data.zip › Supplemental Figure S6.pdf]

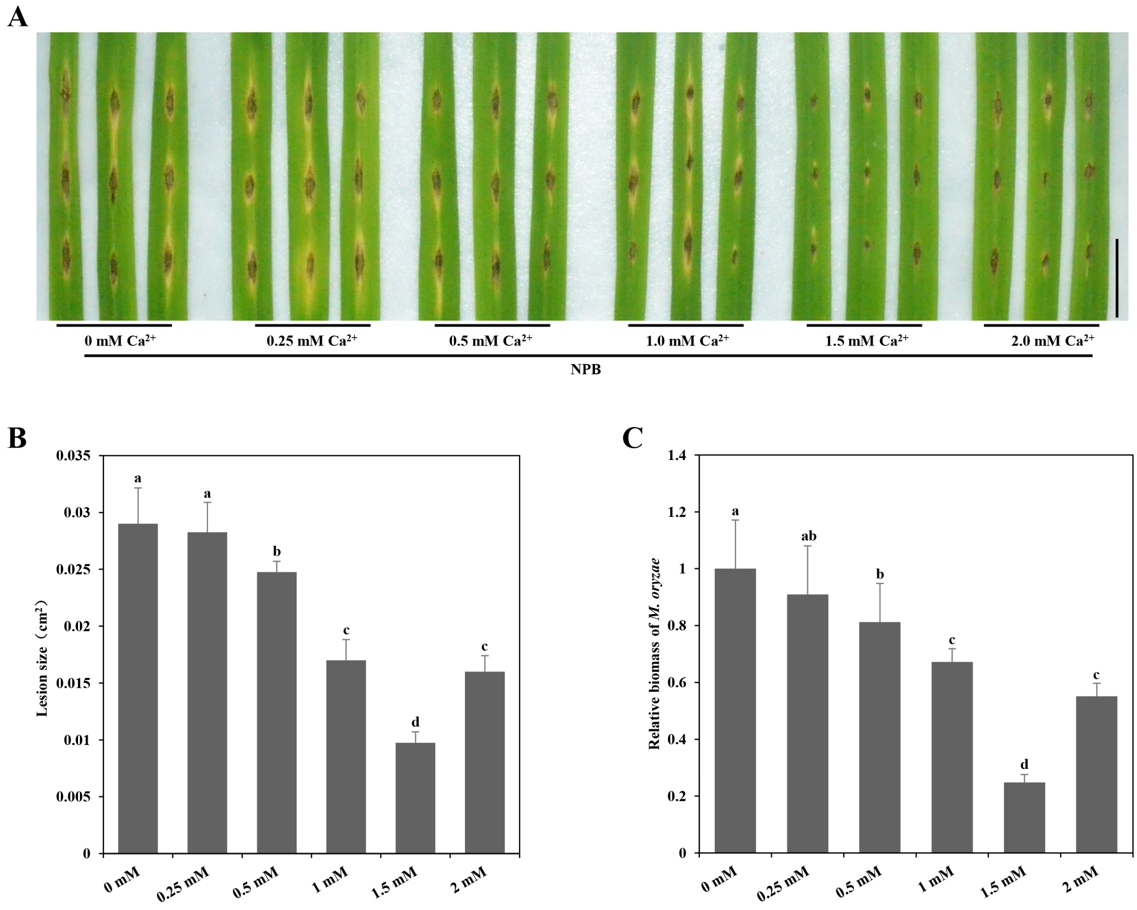

Supplemental Figure S6. Effect of gradient external Ca^{2+} on disease resistance. (A) Phenotype of rice blast disease after adding different concentrations of Ca^{2+} (B) Lesion size (B) and relative biomass of *M. oryzae* (C) of picture (A). Values are means ± SD (B and C, n= 3 replicates). The Student's t-test analysis indicates a significant difference (*$P < 0.05$, **$P < 0.01$).
