# Supplementary material for: Long small RNA76113 targets CYCLIC NUCLEOTIDE-GATED ION CHANNEL 5 to repress disease resistance in rice
Source: Plant Physiol. 2023 Nov 9;194(3):1889–905. doi: 10.1093/plphys/kiad599 (PMC10904327; doi:10.1093/plphys/kiad599)
Supplement: kiad599_Supplementary_Data [file kiad599_supplementary_data.zip › Supplemental Figure S7.pdf]

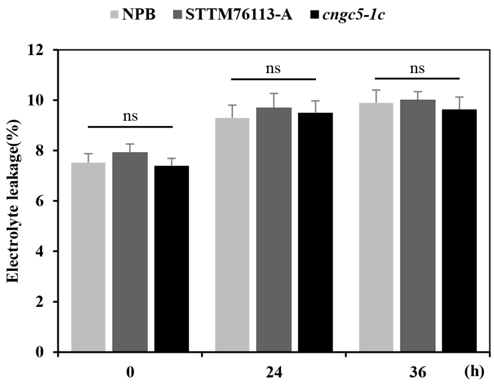

Supplemental Figure S7. OsCNGC5 does not affect rice ETI response. Values are means ± SD (n= 3 samples). The Student’s t-test analysis indicates a significant difference (*P < 0.05, **P < 0.01). ns, Not Significant.
